# Acknowledgment to the Reviewers of *Animals* in 2022

**DOI:** 10.3390/ani13040659

**Published:** 2023-02-14

**Authors:** Animals Editorial Office

**Affiliations:** MDPI AG, St. Alban-Anlage 66, 4052 Basel, Switzerland

High-quality academic publishing is built on rigorous peer review. *Animals* was able to uphold its high standards for published papers due to the outstanding efforts of our reviewers. Thanks to the efforts of our reviewers in 2022, the median time to first decision was 18 days and the median time to publication was 43 days. Regardless of whether the articles they examined were ultimately published, the editors would like to express their appreciation and thank the following reviewers for the time and dedication that they have shown *Animals*:
A. N. M. Mamun-Or-RashidKathiresh ManiAage Kristian Olsen AlstrupKathleen ColegroveAbdallah Tageldein MansourKathleen CrandellAbdelmutalab AzragKathleen McEnteeAbdolmohammad Abedian KenariKathleen MorganAbdolreza R. HosseindoustKathleen R. Pritchett-CorningAbdulai GuinguinaKathreen E. RuckstuhlAbdulmojeed YakubuKathryn A. SeabaughAbe HiroyaKathryn E. SeeleyAbel Villa-ManceraKathryn NankervisAbele KuipersKathryn Y. BurgeAbhinandan ChowdhuryKatia CappelliAbigail GazzardKatia PinelloAbiodun Mayowa AkanmuKatie HallAbmael da Silva CardosoKatie Lynn SummersAboelenain MansourKatie McDermottAbouzar NajafiKatie PotterAbraham Méndez-AlboresKatie YoungAbu SayeedKatiuska SatuéAchille SchiavoneKatja M. GuentherAd J. C. De GroofKatja N. KoeppelAda RotaKatja SteigerAdam BurtonKatrin GillerAdam Hartstone-RoseKatrina E. HollandAdam J. DavisKatrina MerkiesAdam MasłońKatsufumi SatoAdam Michael LewisKatsunori OkazakiAdam RomanKatsuro HagiwaraAdam TofilskiKaty FulferAdam WąsKaty SchroederAdam WeitemierKa-Yee Grace ChoiAdam ZiecikKazeem AdeyemiAdebowale AdebiyiKazuharu NomuraAdel PezeshkiKazuhiko HosakaAdela MartinezKazuhiko ImakawaAdela Oliva ChavezKazuhiko OchiaiAdele FabbrociniKazutoshi NishijimaAdele WilliamsKees C. G. van ReenenAdelene WongKegan Romelle JonesAdolfo Maria TambellaKeila Acevedo-VillanuevaAdrian A. BarraganKeisuke SuganumaAdrian A. Díaz-SánchezKeisuke SugimotoAdrián CasanovaKelly A. GeorgeAdrián Felipe González AcostaKelly AnnesAdrian GrozeaKelly GettyAdrian Zaragoza-BastidaKelly KingonAdriana A. Cortés-GómezKelsey Margaret Harvey SchubachAdriana Alonso NovaisKelsy J. RobinsonAdriana BelasKemal Tuna OlğaçAdriana Cecilia PaciniKeni RenAdriana CortezKenichi HaranoAdriana Di TranaKen-ichiro InoueAdriana DomínguezKenji FukudaAdriana FerlazzoKenji KutaraAdriana GradelaKenneth AndersonAdriana GyӧrkeKenneth GenoveseAdriano R. LameiraKenneth H. McKeeverAdrien ButtyKenneth J. GenoveseAdrienne E. CrosierKenneth OnditiAdronie VerbruggheKenneth R. HarkinAftab ShaukatKenneth ShapiroAgata Antoniewska-KrzeskaKenneth W. BafundoAgata BiadałaKensuke TairaAgata Jabłońska-TrypućKerry BartieAgata WawrzyniakKerry HuntAgata ZmijewskaKerstin E. AuerAgata ZnamirowskaKeshun LiuÁgnes CsivincsikKevin AndersonAgnès Perrin-GuyomardKevin D. NiedringhausAgnese BalzaniKevin K. HausslerAgnieszka AntończykKevin Le VergerAgnieszka BiałekKevin McPeakeAgnieszka BieniekKevin Panke-BuisseAgnieszka BlitekKeying ZhangAgnieszka BossowskaKezong QiAgnieszka HerosimczykKhadija AkaridAgnieszka LudwiczakKhajamohiddin SyedAgnieszka Noszczyk-NowakKhaled ItaniAgnieszka NowakKhizar HayatAgnieszka OrkuszKhuliso Emmanuel RavhuhaliAgnieszka PartykaKi Beom JangAgnieszka Perec-MatysiakKi-bae HongAgnieszka Wiszniewska-ŁaszczychKichang LeeAgnieszka Żak-BochenekKim A. BardAgnieszka ZiemiańskaKimberley BennettAgnieszka ŻuryńKimberly Ange-van HeugtenAgostina Zubiri-GaitánKimberly BreitenbecherAgostino SeviKimio FukamiAgustín AriñoKinga Korpiwiec-DomańskaAgustín CamachoKiriaki PavlidouAgustín Olmedo-JuárezKirrilly ThompsonAharon R. FriedmanKirsten Marie Bay TjørnehøjAhmad HamedyKirsten PerssonAhmad Jawad SabirKirstin Dahl-PedersenAhmed A. SalehKirsty LeśniakAhmed Abdel-WarethKiwamu HanazonoAhmed Eisa ElhagKiyonori KawasakiAhmed ElolimyKnud LarsenAhmed KheimarKo-Hua TsoAhmed MustafaKoichi HagiyaAhmed S. MandourKollur Shiva PrasadAhmet AltindagKomala ArsiAigües Repullés-AlbeldaKondoh DaisukeAime K. JohnsonKonrad FischerAinhoa AgorretaKonrad SachseAinhoa Sarmiento-GarcíaKonstantinos ArsenopoulosAinhoa ValldecabresKor OldenbroekAishwarya MaheshwariKorawan SringarmAitor Fernandez-NovoKorinna HuberAitor Garcia-VozmedianoKornel KasperekAjitanuj RattanKosmas VidalisAkane TanakaKostas SagonasAkanksha SinghKouichi KawamuraAkansha M. ShahKovy Arteaga-LiviasAkebe Luther King AbiaKowalska DorotaAkhilesh Kumar VermaKris H. SabbiAkinori YamadaKris HineyAkira IshikawaKrishnamoorthy SrikanthAkira YabukiKristen BradyAkiyoshi TakahashiKristene R. GedyeÁkos BodnárKristian Ellingsen-DalskauÁkos GellértKristie CameronÁkos JerzseleKristijan TomljanovicAl VrezecKristin A. JonassonAlaa Emara RabeeKristina HorbackAlaa MansourKristina MuellerAlain FontbonneKristina TvarozkovaAlan K. OutramKristine HillAlan LillKristjan BregendahlAlan M. YoungKristyn R. VitaleAlasdair J. NisbetKrisztián BaloghAlassane KeïtaKrisztián FrankAlba GalafatKrisztián KatonaAlbert J. AugusteKritapon SommartAlbert Johannes BuitenhuisKrizler TanalgoAlbert MontoriKrystyna CwiklinskiAlberta De StefaniKrystyna Pierzchala-KoziecAlberto ArencibiaKrzysztof AdamczykAlberto Barreras-SerranoKrzysztof AndresAlberto CesaraniKrzysztof AnuszAlberto CollaretaKrzysztof DamaziakAlberto ContriKrzysztof FlisikowskiAlberto ElmiKrzysztof KowalAlberto Menendez BuxaderaKrzysztof KuprenAlberto MeriggiKrzysztof LipińskiAlberto PrandiKrzysztof RypułaAlberto PrietoKrzysztof ŚmietankaAlcira Ofelia DíazKrzysztof TomczukAldo CorrieroKumar AmitAldo VezzoniKumbukani MzengerezaAldona KawęckaKun-Ling TsaiAleena JoyKuo-Wei LanAlejandra Garcia-GascaKurt FristrupAlejandro Casanova-HigesKurt G. M. De CramerAlejandro J. Marquez-LaraKurt HammerschmidtAlejandro PlascenciaKurt KotrschalAlejandro Suárez-PérezKurtis Jai-Chyi PeiAlejandro Zamora RestanKwang-Hyeon ChangAlek RachwaldKwangwook KimAleksandar CojkicKwanseob ShimAleksander SzczurkowskiKyle A. GarverAleksandra Alicja DrażboKyle LovercampAleksandra BiedrzyckaKyle McLeanAleksandra CebulskaKylen GartlandAleksandra CybulskaKylie CairnsAleksandra DunisławskaKyohei FurukawaAleksandra Górecka-BruzdaKyoko Tsukiyama-KoharaAleksandra KizhinaKyoung Seong ChoiAleksandra ŁangowskaKyran E. KunkelAleksandra PawlakKyung-Woo LeeAleksandra PetrovićL. Di StasioAleksandra Pliszczak-KrólLabrini V. AthanasiouAleksandra TroscianczykLaiba ShafiqueAleksandra UzelacLaibané Dieudonné DahourouAleksandra ZgrundoLaila DarwichAleksandro Schafer Da-SilvaLaila Muñoz-CastellanosAlen DžidićLaima BalčiauskienėAlena PechováLainie CameronAlenka LevartLakamy SyllaAleš LapanjeLanre MorenikejiAleš PavlíkLarry CarboneAlessandra AlessianiLarry CroftAlessandra CrisàLarry L. BowmanAlessandra FerramoscaLars Erik RuudAlessandra GaffuriLars F. H. TheyseAlessandro BalestrieriLars HillströmAlessandro BellatoLars Olav EikAlessandro BenedettoLászló BozóAlessandro Dal BoscoLászló EgyedAlessandro F. T. AmaranteLászló FodorAlessandro FeolaLászló SzemethyAlessandro FerrariniLaudí Cunha LeiteAlessandro FiorettiLaura A. AdamoviczAlessandro GramenziLaura A. FavettaAlessandro GuerriniLaura A. ReeseAlessandro MarroneLaura Abril-ParreñoAlessandro PirasLaura B. HeilenAlessandro PoliLaura BenitezAlessandro ReggianiLaura BortolottiAlessandro RicciLaura BruneauAlessandro SpadariLaura CalvilloAlessandro StamillaLaura ContalbrigoAlessandro VastoloLaura DixonAlessia DianaLaura EdwardsAlessia LucianiLaura GrahamAlessia MariacherLaura GreinerAlessio AlesciLaura GribaldoAlessio PieriniLaura HänninenAlessio ScarafoniLaura Helen KramerAlex D. GreenwoodLaura K. MarshAlexander A. KirillovLaura KvistAlexander DvoretskyLaura López-GrecoAlexander GatesLaura M. KubasiewiczAlexander GorbushinLaura MenchettiAlexander GrahoferLaura MusaAlexander KrivoruchkoLaura Núñez-PonsAlexander S. HallLaura OzellaAlexander TavellaLaura Patterson RosaAlexander TiniusLaura RibeiroAlexander V. SirotkinLaura Teresa Hernández-SalazarAlexander WerthLaura WhalinAlexander YitbarekLaura X. Estévez-MorenoAlexandra C. GreenLaure GatelAlexandra E. FranciscoLaurel E. K. SerieysAlexandra EconomouLauren AugustineAlexandra GambaryanLauren FinkaAlexandra H. A. DugdaleLauren HannaAlexandra J. MalbonLauren PowellAlexandra LenglingLaurence ChèzeAlexandra PalmerLaurent BigarréAlexandra WealleansLaurent DeloozAlexandre AzevedoLaurent HébertAlexandre M. PinheiroLaurent LocquetAlexandre PimentaLaurent TiretAlexandre Rodrigues SilvaLav BavčevićAlexandros KougioumtzisLavanya GoodlaAlexandros MavrommatisLavinia ChitiAlexandros TheodoridisLaxman KhanalAlexandru GudeaLea RempelAlexandru VulpeLeandro CelestinoAlexey PiskunovLeandro M. VieiraAlexey RuchayLech AdamczakAlexis Ruiz-GonzálezLech KirtiklisAlexsandra Fernandes PereiraLee Allen PetteyAlfonso Aguilar-PereraLee Seong WeiAlfonso BolarínLee-Anne HuberAlfonso ZecconiLeena AwawdehAlfredo AttisanoLei HuAlfredo E. S. de BorbaLei ZhouAlfredo Estrada-AnguloLeif K. RasmusonAlfredo González-GilLeilson Rocha BezerraAlfredo Jorge Costa TeixeiraLena LutzAlfredo PauciulloLene KleppeAlfredo Quites AntoniazziLenny van Erp-van der KooijAlfredo RamírezLeo J. CumminsAlgimantas PaulauskasLeon J. BroomAli Adem BaharLeonardo AncillottoAli AygunLeonardo De Oliveira SenoAli Emre OzturkLeonardo MeomartinoAli ForouharmehrLeonardo Nanni CostaAli HamidoghliLeonardo SáenzAli RostamiLeonel Herrera AlsinaAliai LanciLeonie JacobsAlica PizentLeontides LeonidasAlice BrandãoLeopold Slotta-BachmayrAlice FerrariLeopoldo PérezAlicia EstevezLesley A. HawsonAlicja BorońLeslie IrvineAlicja Dratwa-ChałupnikLetizia FiorucciAlicja KowalczykLev YampolskyAlina Pyka-PająkLevent BatAlireza SeidaviLevent GizemAlison GardnerLevente CzeglédiAlison R. GerkenLi ChangAlison SmallLi GuoAlison WillsLiam ClayAlistair LegioneLiang ChenAlistair Victor William NunnLiang Chou HsiaAlkiviadis VatopoulosLiang XieAlla KrasikovaLiara GonzalezAllan L. SchaeferLiča LozicaAllan StrongLida FekratAllen CollinsLídia Colominas BarberàAllen T. RutbergLidia Lasecka-DykesAllison AndrukonisLidija PericAllison MartinLiew Hon JungAllison PullinLifei WangAlma D. Alarcon-RojoLiga WuriAlois CizekLigang WangAlvaro BicudoLiisa VeerusÁlvaro Efrén Domínguez RebolledoLilach GavishÁlvaro José De Almeida BicudoLiliana Aguilar-MarcelinoÁlvaro MourenzaLiliana SousaAlyssa A. LoganLily ParkinsonAmali U. AlahakoonLin Lee-ShuanAmanda BradberyLina María Martínez-LópezAmanda EmburyLina RothAmanda GibsonLinan JiaAmanda HaleLinas BalčiauskasAmanda K. Warren-SmithLinda BesterAmanda LindholmLinda D. BakerAmanda Marchi MaioranoLinda J. WaltersAmaranta de Miguel-RubioLinda Jean LavenAmber Adams-ProgarLinda NeavesAmber BarnesLindsay FryAmber LeeLindsay G. A. McKayAmélia Camarinha-SilvaLindsay HamiltonAmelia G. WhiteLindsay M. FryAmely CampeLindsay MarshallAmin SayyariLindsay MatthewsAmina ZuberiLindsay St. GeorgeAmir FallahshahroudiLindsey Marie VansandtAmir Mahboubi SoufianiLindsey RobbinsAmit KumarLingling JinAmiya Kumar SahooLinh NguyenAmke CaliebeLinjun HongAmlan Kumar PatraLinyong HuAmna SaharLinyuan ShenAmnart PoapolathepLionel SebbagAmparo Ruíz-SauríLíriahiromi Hiromi OkudaAmr Abd El-WahabLisa Adele PirasAmulya YaparlaLisa BachmannAmy BarstowLisa CliffordeAmy DesaulniersLisa EkmanAmy FultzLisa FlamminiAmy JohnsonLisa HolmesAmy L. MacNeillLisa JungAmy LockwoodLisa M. RileyAmy MillerLisa Maria GlenkAna Alvarez VellosoLisa MehlmannAna Canadas-SousaLisanework AyalewAna Carolina S. ChagasLise Doksæter SivleAna Catarina TorresLiu LeiAna Catarina Vieira Vieira De CastroLiudmila LysenkoAna Clara MenezesLiv BakerAna Claudia CarreiraLivia MoscatiAna Cláudia CoelhoLivio GalosiAna Cristina Silvestre FerreiraLiyuan CaiAna Gomes-BispoLleretny Rodríguez AlvarezAna HuertasLloyd A. CourtenayAna I. PeñaLluis Ferré-DolcetAna I. ReyLluis LujanAna Isabel Garcia-RuizLong GuoAna Isabel PrietoLong ShengAna Isabel Roca-FernándezLong ZhangAna IvanovićLonghui ZhaoAna JimenezLoredana BasiricoAna Lúcia M. YamadaLorella GiuliottiAna M. SahagunLorella ManiscalcoAna MagalhãesLorella NotariAna Manuela De AzevedoLorena Garcia-del RioAna Maria BenedekLorena Jiménez SobrinoAna MuñozLorena VarrialeAna Paula BastosLorendane Millena De CarvalhoAna Pérez-CembranosLorenzo DomenisAna Rita PatricioLorenzo LesoAna SantanaLorenzo ServaAna Shek-VugrovečkiLorenzo TiduAna Silvia Alves Meira Tavares MouraLori K. SheeranAnabela MoreiraLori R. KoganAnalía del Valle GarneroLorna CameronAnalivia BarbosaLouis ChoncoAnamaria LazărLouis WeissAnand SinghLouise BuckleyAnanda FelixLouise M. SoanesAnastasia DiakouLouise ReillyAnastasia Morandi-RaikovaLouise van der WeydenAnastasia VassettiLourdes Mateos-HernándezAnat Shnaiderman-TorbanLourens J. ErasmusAnca GheorgheLuboš VostrýAnderson de Moura ZanineLuboš ZábranskýAndra-Elena EnacheLuc MaertensAndrás GáspárdyLuca CiceroAndre BogevikLuca De SabatoAndre C. MorandiniLuca FontanesiAndré Guimarães Maciel SilvaLuca IlahianeAndré L. V. ZoppaLuca LapiniAndre Rodrigues ViannaLuca Maria BattagliniAndré Thaler NetoLuca SardiAndré V. RubioLuca VillaAndrea BertugliaLucas BlantonAndrea BragaglioLucas BoerAndrea CordaLucas Dos Santos DiasAndrea De FeliceLucas HallAndrea Dorothea EllisLucas M. LeveauAndrea FulgioneLucía Alarcón-RíosAndrea GamaLucia AmendolaAndrea Gudan KuriljLucia BailoniAndrea I. Moreno-SwittLucia CarlucciAndrea M. HarveyLucia CondorelliAndrea MarchegianiLucia Giuliano CaetanoAndrea MünnichLucia MartinezAndrea PaoliniLucia MinoliAndrea RandoLucía Perez-PardalAndrea Rita MarreroLucia RocchiAndrea SerraLucia RoccoAndrea SummerLucia ViñasAndrea SvoradováLuciana B. da CostaAndrea VivianoLuciana CastaldoAndrea ZatelliLucianna MaruccioAndreana PexaraLuciano MorbidiniAndreas BertsatosLuciano PinottiAndreas G. TsantesLuciano Soares De LimaAndreas HoppeLucio PetrizziAndrei Cristian GradinaruLucjan WitkowskiAndreia C. M. RodriguesLucrecia Souviron PriegoAndreia GarcêsLucy A. BatesAndreia SantosLucy Bearman-BrownAndrés GambiniLucyna KirczukAndrew AllenLuděk BartošAndrew ButterworthLudmila KřížováAndrew C. GallupLudovica ChiavacciniAndrew DalbyLudwig E. HoelzleAndrew E. DerocherLuiene M. RochaAndrew G. GoslerLuigi BonizziAndrew GehringLuigi GalloAndrew J. K. ConlanLuigi NavasAndrew KnightLuigi VencoAndrew Nicholas RowanLuigi ZicarelliAndrew NicolLuis Ángel Rubio San MillanAndrew NiehausLuis Ángel Zarazaga GarcésAndrew PetersLuís BonifácioAndrew R. HalloranLuís CardosoAndrew S. McPhersonLuis Corona GochiAndrew ShawLuis CubillosAndrew ShedlockLuis David Solis MurgasAndrew W. ByrneLuis J. Ezquerra CalvoAndrey ChernovLuis J. RoyoAndrey GoginLuis LlanezaAndrey MukhinLuís LoboAndrey ShirakLuís Madeira de CarvalhoAndrzej DybusLuis Perez Garcia-EstañAndrzej GugołekLuis QuintelaAndrzej OkruszekLuis Ramirez-AvilesAndrzej ZachwiejaLuis Sarmiento-FrancoAndrzej ZybertLuis Vicente MonteagudoAndy HerringLuisa GarofaloAndy Wai Kan YeungLuisa MagrinAneela Zameer DurraniLuisa RambozziAneliya MilanovaLuisa ValentiniAnet Spengler NeffLuisa Vera MuscatelloAneta NowakiewiczLuiz Daniel de BarrosAnette WichmanLuiz Henrique P. SilvaAngel Gustavo Salas-LaisLukáš BujňákAngela BarbosaLukas SchwarzAngela BergschmidtLukas VeselyAngela CostaLukáš ZitaAngela Di CesareŁukasz AdaszekAngela FaddaŁukasz KajtochAngela FournierŁukasz MigdałAngela HollandŁukasz TomczykÁngela L. DebenedettiŁukasz WlazłoAngela LaveryLuke IwanowiczÂngela MartinsLuke Mangaliso DuncanAngela Palumbo PiccionelloLuke O’GradyAngélica da Silva VasconcellosLurdes ClementeAngélica María Sánchez-SarmientoLuyu DingAngélica Villarruel-LópezLyda G. GarciaAngelika SchosterLydia LanzoniAngeliki KatsafadouLydia Meriel HopperAngelo GazzanoLynette A. HartAngelo QuarantaLynn M. PezzaniteAngus M. A. ReidLynne SneddonAniello FrancescoLyudmila KhrabrovaAnil SigdelM. A. ArcangioliAnindya Sundar RayM. C. S. OliveiraAnisa RibaniM. Dulce EstêvãoAnita E. FlemingM. L. SantanaAnita Mitico Tanaka-AzevedoM. Susan DeVriesAnita OberbauerM. T. TejedorAnja PalandačićMaamer JlaliAnja SipkaMaaria KortesniemiAnkush PrasadMaarten OosterlinckAnn HemingwayMacdonald WickAnna Antonella SpinaMaciej FrantAnna BajerMaciej GrzybekAnna BassolsMaciej JaneczekAnna BerghMaciej KamaszewskiAnna Chur-HansenMaciej KochanowskiAnna CywinskaMaciej SkorackiAnna DedousiMaciej SzewczykAnna DidkowskaMaciej ZdunAnna DziekońskaMadhu RaviAnna GagliardoMagdalena Agata GarncarzAnna GoslingMagdalena BuniowskaAnna KaragianniMagdalena GantnerAnna Karolina MatczukMagdalena Garijo ToledoAnna KasprzykMagdalena KolendaAnna Kate ShovellerMagdalena KrauzeAnna KoseniukMagdalena Maria GryzińskaAnna Kristina MykkänenMagdalena MaselloAnna KrutikovaMagdalena Mazur-KuśnirekAnna Lenart-BorońMagdalena MurrAnna ŁepeckaMagdalena PieszkaAnna Luiza Facchetti Vinhaes AssumpçãoMagdalena PodbielskaAnna M. KaczmarekMagdalena Polak-ŚliwińskaAnna Machoy-MokrzyńskaMagdalena SchrankAnna Maria AddamoMagdalena SochaAnna Maria Pérez-VendrellMagdalena SolkaAnna MigdałMagdalena Zatoń-DobrowolskaAnna MilczarekMagdalena ŻmigrodzkaAnna NekarisMaggie NicholsAnna OrtuñoMagnus CamplerAnna RogiewiczMagnus GidlundAnna RząsaMahesh NeupaneAnna SansoneMahlako L. MakgahlelaAnna SawickaMahmoud MadkourAnna ScandurraMahmoud Mostafa AzzamAnna ShepelyakovskayaMailin GanAnna StachurskaMáire Catríona McElroyAnna StępniowskaMaja Ivana Smodiš ŠkerlAnna Szczerba-TurekMaja MiskulinAnna Szuba-TrznadelMaja Zakošek PipanAnna Tus̈nioMajid KhansefidAnna WesselsMajid ShakeriAnna Winiarska-MieczanMakoto SugiyamaAnna WójcikMalcolm JoblingAnna WyrostekMalcolm McAdieAnna WysokińskaMalgorzata C. BehnkeAnna ZamoraMałgorzata Chmielewska-KrzesińskaAnna. J. KorzekwaMałgorzata D. Klimowicz-BodysAnnalisa RizzoMalgorzata DominoAnnalisa VianiMałgorzata DudaAnnamaria CostaMałgorzata GieryńskaAnnamaria PassantinoMałgorzata GolemanAnnamaria PratelliMalgorzata GrzesiakAnnamaria UvaMałgorzata GrzesiukAnn-Chang ChengMałgorzata Kasprowicz-PotockaAnne BergerMałgorzata Korczak-AbshireAnne BlaisMałgorzata Kotula-BalakAnne BonnieuMałgorzata KwiecieńAnne K. SchützMałgorzata MaśkoAnne LaarmanMałgorzata Natonek-Wi’sniewskaAnne M. ForemanMałgorzata OchotaAnne McBrideMałgorzata OżgoAnne MullenMałgorzata P. MajewskaAnne SilvestreMałgorzata PawlosAnne StrailyMałgorzata Pomorska-MólAnneke MorescoMałgorzata ŚwiątkiewiczAnnelies DecloedtMałgorzata SzczesnaAnnelisse CastilloMałgorzata WierzbickaAnnemarié Avenant-OldewageMami MurakamiAnne-Marie LutherManal HemidaAnnesly NetthisingheManasés González-CortazarAnnette KutterManat ChaijanAnnette LiesegangMandar NanajkarAnnette ZeynerMandi CarrAnnik Imogen GmelMandy PatersonAnnika PosautzManfred CoenenAnn-Kristin BartonManfred WeidmannAnoosh RakhshandehManohar KodavatiAnouschka MiddelkoopManoj KumarAnte IvankovićManon HoubenAnthony Pokoo-AikinsManon SchweinfurthAnthony ValverdeMansur Abdullah SandhuAnthony William ColemanManuel AvilesAntigone UzunidisManuel BollerAntón García-MartínezManuel FondevilaAnton KováčikManuel Gonzalez-RonquilloAntonella Della MalvaManuel Gutierrez-AguilarAntonella PuggioniManuel IglesiasAntonella TinelliManuel NovalesAntonella VoltaManuel PizarroAntónia MacedoManuel Ramiro Rodríguez RodríguezAntonín PřidalManuel RamónAntonino CalapaiManuela CrastaAntonino NizzaManuela VilhenaAntonino PaceMar ComasAntonio AlmeidaMar Yllera FernándezAntonio BoscoMarc BoelhauveAntonio BuendíaMarc E. H. JonesAntonio CarminatoMarc GirondotAntonio GiulianoMarcella MassiminiAntonio Gonzalez CantalapiedraMarcella Zampoli TroncarelliAntonio Gonzalez-BulnesMarcello D’AmicoAntonio González-CasadoMarcello TagliaviaAntonio Leandro Chaves GurgelMarcelo A. CatalánAntonio MolinaMarcelo Abidu-FigueiredoAntonio MolloMarcelo AlmeidaAntónio MonteiroMarcelo FerreiraAntonio Palomo YagüeMarcelo GómezAntónio Paulo CarvalhoMarcelo Maia PereiraAntonio SantanielloMarcelo Palma SirciliAntony VincentMarcelo Rodriguez FerrereAnuj AhujaMarcelo Tigre MouraAnusha DhanasiriMarcia FrancoAnusz KrzysztofMarcia H. M. R. FernandesAparna KrishnavajhalaMarcia Mayumi IshikawaAphrodite I. KalogianniMárcia Saladini Vieira SallesApostolos D. GalatosMarcin ArciszewskiApril A. KedrowiczMarcin BarszczAraceli Samaniego HerreraMarcin KucińskiArantza VitoriaMarcin LisArati SridharanMarcin MichałekAravinthkumar JayabalanMarcin RóżewiczArcesio Salamanca CarreñoMarcin SońtaArchana AbhijithMárcio S. DuarteArgenis RodasMarco A. F. LopesArianne MessermanMarco AcciaroArie van NesMarco AlbanoArif CilogluMarco Alberto Luca ZuffiArif RezaMarco ArculeoArif SiddiquiMarco BiroloArimune MunakataMarco BottinelliArinze OkoliMarco CamperaAris PourlisMarco CuccoAristotelis G. LymberopoulosMarco Di DomenicoArkadios DimitroglouMarco GaleottiArkadiusz FröhlichMarco GerdolArkadiusz KajdaszMarco Juárez-EstradaArkadiusz MarciniakMarco MangiacottiArlind MaraMarco MilanesiArmida Sánchez-EscalanteMarco RosatiArmin R. W. ElbersMarco SarogliaArnaud BianchiMarco TecillaArno E. LindnerMarco VasconcelosArnold KanengoniMarco ZeddaÁrpád BajcsyMarcos V. CaiafaArsen DotsevMarcus Antonio ZanettiArthur R. FramptonMarcus Baynes-RockArtur Jorge FerreiraMarcus FelicianoArtur M. RydoszMarek BabiczArtur Severo VarejãoMarek BienkoArun RajamohanMarek GaworskiArunika DasMarek Jan ŁuczyńskiArunima SikdarMarek KotowiczArunkumar RanganathanMarek KowalczykArvind SharmaMarek LecewiczArzu UçarMarek PieszkaAsha M. MilesMaren HuckAshley EdesMaren Ortiz-ZarragoitiaAshley K. PutmanMargalida JoyAshley PrichardMargaret E. GruenAshraf AlkhtibMargaret LeMoneAshwin RameshMargaret SlaterAspinas ChapwanyaMargarita N. FavorskayaAtanu PatiMargherita MarzoniAthanasios RagkosMargret L. CasalAthanasios SamarasMaria AlbrizioAthina BasiouraMarìa Alejandra FortinoAthina S. TzoraMaría Alejandra ParreñoAtsuki NaraMaría Ángeles CalvoAtsushi NanamiMaría Angeles PérezAtta Kofi AgyekumMaria Angeles RojoAttawit KovitvadhiMaria Angelica MiglinoAtte Johannes von WrightMaria CappaiAttila MartonMaria Carmela PisuAttila ZsolnaiMaria Consuelo MuraAttilio CorradiMaria Cristina AngeliciAugusta GasparMaria Cristina BressanAugustine Thomas PeterMaria Cristina CozziAugusto CarluccioMaria Cristina GuerreraAugusto M. L. MadureiraMaria Cristina VeronesiAugusto VitaleMaria da Conceição FontesAurelia LerouxMaria DaskalakiAurélie ManinMaria de Fátima Martins Lorena de OliveiraAurora OrtínMaria de OliveraAusilia GrassiMaría Dea-AyuelaAustin LeedsMaría del Refugio María del RefugioAutchara KayanMaria Del Rosario Rico FerreiraAvinash PremrajMaria DevantAwad ShehataMaria Do Rosário Fernandes MarquesAwal FuseiniMaría Dolores Fernández RodríguezAyaka T. MatsudaMaría Dolores Llobat BordesAyelén Melisa BlancoMaria Eduarda PotesAymen FrijaMaria Elena Trujillo OrtegaAyodeji Simeon AderibigbeMaría Esther Ortega-CerrillaAzbas Am TaurusmanMaria Esther RodriguezAzger DusthackeerMaria Eugênia Andrighetto CanozziBabak DarabighaneMaria Eugenia Lopez-ArellanoBahar PatlarMaría Florencia GallelliBai XueMaria Font I. Font I FurnolsBailey EngleMaria Giovanna CilibertiBailey HarshMaria Gracia GervasiBal Krishna ChaubeMaria I. MüllerBalamuralikrishnan BalasubramanianMaría J. DelgadoBalasubramanian SenthilkumaranMaria J. Vilar AresBao YuanMaría Jésus Esús ZamoranoBaofeng SuMaria João PeixotoBaosheng ZengMaria João SantosBarakaeli Abdieli NdossiMaria João SoaresBarbara Borawska-JarmulowiczMaria Jorge Geraldes CamposBarbara BrunettiMaría José García-IglesiasBarbara ColittiMaria Jose Sanchez GuerreroBarbara ContieroMaría José Sánchez-GuerreroBarbara de MoriMaria José T. Ranzani-PaivaBarbara DurrantMaría Josefa Fernández-del PalacioBarbara HinneyMaria KantereBarbara JonesMaria Laura MarongiuBarbara KowalikMaria Lúcia Gomes LourençoBarbara KrolMaria Luisa DettoriBárbara Martín-MaldonadoMaría Martín-CuervoBarbara MerloMaria MastorakiBarbara MoroniMaria Maust-MohlBarbara PadalinoMaría Montserrat Rivera del ÁlamoBarbara PratscherMaria OczkowiczBarbara ShihMaría PermuyBarbara StefańskaMaria PerottiBarbara SzczepankiewiczMaria QuestaBarbara TombarkiewiczMaria RebordãoBarbara WasilewskaMaria Rodrigues da CostaBarbara WilliMaría Sáez-RoyuelaBarbara WróbelMaria Santina C. MoriniBarbara ZoricaMaria Susana LopesBartlomiej JaskowskiMaria Teresa Armúa-FernándezBartosz BojarskiMaria Teresa CapucchioBartosz BorczykMaría Teresa DíazBartosz KierończykMaría Teresa Gómez-MuñozBartosz PawlińskiMaria Teresa ManfrediBasanta Kumar DasMaria Teresa PacchioliBasilio RandazzoMaría Teresa ParamioBeata GrzegrzółkaMaria VanoreBeata Hukowska-SzematowiczMariá Villalba-OreroBeata KuczyńskaMaria Zelinda RomanoBeata KurowickaMaria-Anastasia KaratziaBeata SzostakowskaMaría-José ArgenteBeata WodeckaMarialaura CorrenteBeata WysokMaría-Luz GarcíaBeatrice Simona KelemenMariam PascualBeatriz BrenerMarian BrzozowskiBeatriz C. D. CuyabanoMarian CzaudernaBeatriz IsabelMarian FlisBeatriz Martínez-VallespínMarian Stamp DawkinsBeatriz Serrano-PérezMariana AlmeidaBeatriz SoretMariana B. MenegatBeatriz WillinkMariana BarbagianniBede F. R. DaviesMariana BentoselaBefekadu ChemereMariana CarriquiryBegoña M. EscribanoMariana CiacciarielloBegoña M. JugoMariana Côrtes BoitéBegoña PaneaMariana Diel de AmorimBekir KabasakalMariana Pedernera RomanoBektaş SönmezMariana PetkovaBelén CuervoMariana SponchiadoBenedetta PasseriMariangela AlbertiniBenedetto SicuroMariangela CaropreseBenigno ElviraMarianna OteriBenito Soto-BlancoMarianna SantonastasoBenjamin BeltzungMarianne FreemanBenjamin C. SmithMarianne M. Sloet van Oldruitenborgh-OosterbaanBenjamin CullMarianne RaithBenjamin D. HoffmannMarianne WernerBenjamin LecorpsMariano PauselliBenjamin TsangMaricy Ferreira ApparícioBenjamin ZwirzitzMarie-Caroline LecrenierBenoit St PierreMarie-Jose Enders-SlegersBeob Gyun KimMarie-Pierre Létourneau-MontminyBernadette O’BrienMarija DuvnjakBernard TurekMarijana VučinićBernd HoffmannMarijke AluwéBernd TellhelmMarilena BazzanoBernhard F. GibbsMarilena BolcatoBert DriessenMarilisa NovaccoBertil BorgMarilyn NorconkBertrand L. DeputteMarin TortiBeth Ann VenturaMarina CerusoBeth ClevelandMarina CordsBethaney TurnerMarina DiioiaBethany Dado-SennMarina Durán-AguilarBethany L. KrebsMarina NeofytouBettina SeegerMarina PaolucciBettina WollankeMarina PiscopoBetty McGuireMarina SpinuBhupinder SinghMarino PrearoBhuwan KashyapMario Alberto Flores-ValdezBiagio D’AnielloMario Alberto Galviz EspinozaBianca Mendes MacielMario BarattaBilal CemekMario CinoneBilal MirMario D’IncauBill BatemanMario Mollo NetoBilly Nzau MatondoMario VenezianiBin LiMariola BochniarzBin LiangMariola SłowińskaBirbal SinghMarion E. EnglandBirgit SpindlerMarion MuchaBirgit U. StetinaMarion PapasBishnu BhattaraiMarion RyanBishnu Prasad DevkotaMarisa ErasmusBlandine DavailMarisa PalazzoBlanka OrłowskaMarisanna SperoniBo Johannes BurlaMaristela PeckleBo WangMárius Vicent Fuentes i FerrerBob ColemanMariusz Pawel KowalewskiBodil Ström HolstMariusz SzmytBogumila PilarczykMariusz TrytekBojana KokićMark BernsteinBoleslaw T. KarwowskiMark BowenBolívar Samuel Sosa-MadridMark F. WiserBonnie BeaverMark FarnworthBonnie ValgaerenMark HansBony D. de KumarMark MasinoBorbala ForisMark R. SandfossBossima Ivan KouraMark S. BoyceBostan NazishMark StewartBoštjan PokornyMark WeaverBo-Wen ShenMarkéta JanovcováBo-Young ChoeMarko AnđelkovićBożena KiczorowskaMarko HaloBożena KróliczewskaMarko KassBożena Nowakowicz-DębekMarko OcepekBožidar RaškovićMarko R. CincovićBožidarka Markovic̈Marko SamardžijaBraden J. CampbellMarkus BastirBrankica MravinacMarkus BaurBranko SuvajdžićMarkus KuehnBreda Jakovac-StrajnMarkus NeuditschkoBrenda E. BallacheyMarkus SchmidBrenda M. AlexanderMarlene Bravo-ParraBreno FragomeniMarlene Katharina KirchnerBrent CredilleMarlyn Hellen RomeroBret A. MooreMarta Barahona MarcoBrett A. DeGregorioMarta BasiagaBrian EllisMarta BorgiBrian J. RudeMarta CanutiBrian McCarthyMarta HenklewskaBrian NielsenMarta HerreraBrianne A. AltmannMarta HerveraBrigid Victoria TroanMarta I. Miranda CastañónBrigitta WichertMarta Inés Saloña-BordasBrigitte BraschlerMarta J. SiemieniuchBrijesh YadavMarta KepinskaBrittany Danielle M. GrahamMarta KiezunBrittany L. BackusMarta LaranjoBrittany WatsonMarta López AlonsoBrittni P. LittlejohnMarta LourençoBrock R. McMillanMarta MarszałekBruce Alexander SchulteMarta MendelBruce RathgeberMarta Parzeniecka-JaworskaBruce W. ChristensenMartha Angélica Gutiérrez-AguirreBruna Calvo AgustinhoMartha OliveraBruna CarbasMartha Smith-BlackmoreBruna MionMarthe Kiley-WorthingtonBrunella RestucciMartijn F. L. DerksBruno J. R. GregórioMartin BachmannBruno RonchiMartin CeballosBruno StefanonMartín FragaBruno TiloccaMartin KöhneBrygida Manikowska-ŚlepowrońskaMartin O. FurrBryony S. TuckerMartin PtacekByung-Jae KangMartin RemmeleCacciola Nunzio AntonioMartin RichterCaio Abércio da SilvaMartín RodríguezCaio Seiti TakiyaMartin SchulzeCaitlin E. O’Connell-RodwellMartin SoustCaitlin GaborMartin SserwaddaCaitlin GrantMartin WittCaiye ZhuMartina ColomboCaleb ReichhardtMartina CrociatiCalin GhermanMartina Francesca MarongiuCalla M. GoekeMartina MiluchováCallum George DonnellyMartina TarantolaCamie HeleskiMartina TorricelliCamila Infantosi VannucchiMartine MeunierCamilla BertoliniMarty RhoadesCamilla MunsterhjelmMartyna BatorskaCamilla SmoglicaMartyna GrzegorzekCanchao YangMartyna KaselaCandice P. ChuMartyna WilkCanwei XiaMarwa IbrahimCari HearnMary Ann McCrackinCarina VisserMary TrachselCarine ElkhoraibiMary WickstenCarine SavalliMaryane Sespere OliveiraCarissa M. KraneMary-Louise PenrithCarl Leo ThurmanMaryse DeleheddeCarl TraeholtMarzena Mogielnicka-BrzozowskaCarla Forte Maiolino MolentoMarzena Rola-ŁuszczakCarla Giuditta VecchiatoMasahiro HayashiCarla IppolitiMasahiro SugimotoCarla LuciniMasahiro YamasakiCarla Maris Machado BittarMasahiro YasudaCarla PalmaMasahito HiguchiCarla PeregoMasami MorimatsuCarleigh E. FedorkaMasamine TakanosuCarles AlcarazMasaru OkudaCarlindo RodriguesMasashi SekinoCarlo BibbianiMasashi YukiCarlo CinqueMasaya IgaseCarlo CorinoMasayasu TaniguchiCarlo CosentinoMasayuki SakataCarlo D’AscenziMasayuki TanakaCarlo GuglielminiMasindi MphaphathiCarlo MengucciMasoud ShiraliCarlo SiracusaMassimiliano TursiCarlo Vittorio CitterioMassimo AmadoriCarlos Aguilar-PérezMassimo BiagettiCarlos Alberto Antunes ViegasMassimo de MajoCarlos Alberto e Silva VenâncioMassimo FaustiniCarlos Alfonso Alvarez GonzálezMassimo GiangasperoCarlos Arriaga-JordanMassimo GiovannottiCarlos Daniel Gornatti-ChurriaMassimo GiuntiCarlos Eduardo Fonseca-AlvesMassimo ZeraniCarlos Eurico Dos Santos FernandesMassinissa YahiaCarlos Garces NarroMasuda YoshitsuguCarlos GrauMateus J. R. Paranhos da CostaCarlos Gutiérrez GutiérrezMateus P. GionbelliCarlos Iglesias PastranaMateusz RawskiCarlos José Dias PereiraMatheus De Paula ReisCarlos M. Becerril-PérezMatheus Gomes da CruzCarlos Palacios RiocerezoMatías Gastón PérezCarlos Pérez-MarínMatilde LombarderoCarlos PiñeiroMatt AkinsCarlos RosasMatt GuilleCarlos S. GalinaMatteo BarbariCarlos Sandoval CastroMatteo MezzettiCarmel NottleMatteo OlimpoCarmela DonatoMatteo OliveriCarmela TripaldiMatteo ZarantonielloCarmela ValastroMatthew BrunkeCarmelo IariaMatthew D. JohnsonCarmelo Ortega RodríguezMatthew Thomas BrewerCarmen BarcenaMatthias KropfCarmen ClappMatthias SchneiderCarmen GalloMatthieu AuthierCarmen JerryMatthieu VignesCarmen MarescaMatthijs MetselaarCarmen MatásMattia BasiliCarmen Nuñez-LahuertaMattia PiroloCarmen Rojas-MartínezMaud BonatoCarmine FinelliMaud ContrantCarol A. GrayMaureen KelleherCarol Chitko-MckownMaureen MacNamaraCarol GrayMaureen PurcellCarola Fischer-TenhagenMaurice L. EastridgeCarolin AdlerMauricio Laterça MartinsCarolina BremmMaurizio MargaglioneCarolina Tamargo de MiguelMaurizio MonaciCaroline ChaucheMauro FasolaCaroline FossumMaxim V. VinarskiCaroline FreyMaxwell Kwame BoakyeCarolyn KingMaya GuptaCarrie J. FinnoMaya MeestersCasey DroschaMaya S. KummrowCaspar WenkMayra M. Gómez CarpioCassandra NuñezMayumi FujiwaraCatalina Rey-ValeirónMazdak SalavatiCatarina EiraMazen BahadiCatarina MilhoMd Elias UddinCaterina Di BellaMd. Abdus SoburCaterina SquillaciotiMd. Amzad HossainCatherine A. RichterMd. Saiful IslamCatherine BaxevanouMd. Tanvir RahmanCatherine BellMechthild Ladwig-WiegardCatherine CarrilloMechthild WiegardCatherine HobaiterMeg EdwardsCatherine J. WalshMegan L. van EmonCatherine TipladyMegan R. LaFolletteCatherine TylanMegan VerdonCathrin PfaffMegan WilsonCathrynne HenshallMeghan T. RamosCathy A. Johnson-DelaneyMeghann PierdonCátia MarquesMehmet Akif BozCátia PacíficoMehmet BozkurtCaven Mguvane MnisiMehmet ErmanCécile Bienboire-FrosiniMehmet GültasCecilia VulloMehmet KizilaslanCecilie MejdellMehmet Ulaş ÇinarCecilio José BarbaMehr NisaČedomir RadovićMelania C. López-CastroCeferino LópezMelania GiammarcoCelia AbolnikMelanie GrahamCeline Cruciani-GuglielmacciMelanie SchärenCéline SterMelinda MerckCesar A. Rosales-NietoMelinda PaholcsekCésar Ocasio-VegaMélissa DuplessisCesar Rosales NietoMelissa HuntChad SchmiedtMelissa J. BainChadi ToumaMengyang FengChaehwa RyuMenno HolzhauerChaiyapas ThamrongyoswittayakulMeredith J. BashawChaiyaphum BunchasakMerel Ritskes-HoitingaChalong WachirapakornMeriel Moore-ColyerChalotte GlintborgMetha MeetamChandan JhaMette S. HerskinChang Eon ParkMezzomo RafaelChang-Bae KimMicaela SgorbiniChang-Jun ZengMichael AzainChanglu WangMichael B. HildrethChangqi LiuMichael BrauchleChaoqun YaoMichael BrücknerChaozheng ZhangMichael CourtCharalampos SiristatidisMichael D. MacNeilCharis LiapiMichael DaviesCharissa HarrisMichael E. BarnesCharlene HanlonMichael HässigCharles El-HageMichael HennessyCharles LiMichael J. AndresCharles SnowdonMichael J. BentonCharles W. ArmitageMichael J. McclureCharlotte Amdi WilliamsMichael KreuzerCharlotte MisbachMichael KubaCharlotte RobinsonMichael LierzCharlotte WinderMichael LindingerChatchote ThitaramMichael McLoughlinChatsirin NakharuthaiMichael MullanChelsea DavisMichael PardoChen-Chih ChenMichael PetersonChenghao ChenMichael PhelpsChengli HouMichael SchutzCheng-Ming TsaoMichael W. FoxCheng-Yih HongMichael WalshChensiang NgMichael WellingtonCheryl KnottMichaela GibsonCheryl LohrMichail KmiecikCheryl MorrisMichał BednarskiCheuk Lun LiuMichał BudkaChi Keung LamMichał BulcChiara AdamiMichał Da̧browskiChiara del PreteMichał DzięciołChiara Di PancrazioMichal GáborChiara GarbarinoMichał GesekChiara Grazia de CiucisMichał ŁobaczChiara LocatelliMichal RolinecChiara Maria Lo FeudoMichal SkibniewskiChiara ScopaMichela BertolaChie Tamamoto-MochizukiMichela BulloneChieh-Yu PanMichela Maria DimuccioChika C. OkaforMichela PuglieseChika TadaMichela RossiChing-Hua TingMichele CostantiniChinh Thi My DamMichele FacciaChinkai ChuangMichele MillerChi-Young WangMichele PazzolaChloe H. StevensMichelle GarciaChloé VigliottiMichelle JendralChong Leong PuanMichelle OsbornChoo Hock TanMidori SakuraChris DickmanMiguel A. AparicioChris JohnsonMiguel A. SilvestreChris MartinMiguel Ángel Hurtado OlivaChris R. PaveyMiguel Angel RamirezChris ShepherdMiguel Ángel Reyes-LópezChris W. RogersMiguel FerrerChristian BöttgerMiguel Gómez-BoronatChristian Cortés-RojoMiguel Henrique de Almeida SantanaChristian DürnbergerMiguel LizanaChristian HanzenMiguel OrtegaChristian KochMiguel QuaresmaChristian PehamMiguel Tavares PereiraChristian VisscherMiguel VelazquezChristiane OtzdorffMihaela NiculaeChristianna HowardMihaela SaracilaChristina BraunMika Asai-CoakwellChristina HarveyMikas IlgūnasChristina MontalbanoMike KingChristina NagelMike McgrewChristina StanleyMikhail ShepelevChristine AurichMikhail Y. SyromyatnikovChristine HägerMiki NakaoChristine Jansen van RensburgMikko NikinmaaChristine KrouzeckyMiklós MézesChristine R. DahlinMilagros Benito HernándezChristine R. RutterMilan K. SharmaChristof Albert BertramMilan KubiatkoChristoph GablerMilan MargetínChristoph RandlerMilena SvobodováChristoph Rüdiger Von BredowMilerky PerdomoChristophe BressacMiles TheurerChristopher A. VarnonMilos JezekChristopher C. PollittMilos VucicevicChristopher C. QuinnMiluše VozdováChristopher CarltonMilyausha KaskinovaChristopher ClarkMin GaoChristopher Darrin HulseyMin HuChristopher J. ByrdMin Jung KimChristopher MichaelsMincheol ChoiChristopher RileyMing DuanChristos AstarasMing SunChrysa VoyiatzakiMingchang HongChrysoula GubiliMingmin LuChryssanthi AntoniadouMingming LiuChuang XuMingwang ZhangChuen-Fu LinMingxia ZhangChun FangMingxing ChuChunfang WangMin-Jin KwakChun-Ming LinMinori ArahoriChun-Ren WangMinoru IkedaChunsheng LiuMinze ZhangChunwang LiMiquel Planas OliverCidéli P. de Paula CoelhoMirabela Oana DumitracheCindy R. ElliserMiranda BertramCinzia GraviliMiranda Mladinić PejatovićCinzia MarchitelliMiranda WorkmanCinzia PiciocchiMirco CorazzinClaire A. WeeksMirella ClausiClaire Beaudu-LangeMirella VazzanaClaire E. CardMiriam Annika VogtClaire Rogel-GaillardMiriam D. GarciaClaire StenhouseMiriam Friedman-EinatClaire WoodMirja C. NolffClara WilsonMirjam SchuchardtClare PalmerMirkka LahdenperäClarisse Simões CoelhoMiroslav JuráčekClaudete R. AlcaldeMirosław BanaszakCláudia AragãoMirosław GrzybowskiCláudia Batista SampaioMirosław KucharskiClaudia CampanaleMirosław RatkiewiczClaudia CastanedaMirosława DabertCláudia Del FavaMirza Dikari KusriniClaudia FugazzaMituru KamiyaClaudia GarridoMitzy Mauthe von DegerfeldClaudia GattaMizuki TaguchiClaudia GiannettoMo D. SalmanClaudia KasperMoez AyadiClaudia KeilMohamed AbdelmegeidClaudia Maria TucciaroneMohamed Abdo RizkClaudia MarquesMohamed Al-SayeghClaudia Mettke-HofmannMohamed DahmaneClaudia OfelioMohamed Rachid AchaabanClaudia Osycka-SalutMohamed Thani IbouroiClaudia PittiglioMohammad Ali HesarinejadClaudia RificiMohammad Ali NilforooshanClaudia SpadavecchiaMohammad DelghandiClaudia WilkeMohammad MoniruzzamanClaudia ZanardelloMohammad NabiClaudio AfonsoMohammed AlzawqariClaudio de LiberatoMohammed Fouad El BasuiniClaudio Iván Serra AguadoMohammed GagaouaCláudio José von ZubenMohammed NaielCláudio Vaz Di Mambro RibeiroMohammed NooruzzamanClaus DeblitzMohammed Rizman-IdidClaus VoglMohammed ZnariClemens FuchsMohammmadreza MohammadabadiClemente López-BoteMohanned Naif AlhussienClotilde B. AngelucciMohd Anuaruddin Bin AhmadonCody L. GiffordMohd Helmy MokhtarColin T. TobinMohd Zamri SaadColline PoirierMokhtar MahouachiCongjun LiMolly C. NicodemusCong-Jun LiMolly NicodemusConny TurniMonali NandyMazumdarConor O’HalloranMoncef ChouaibiConstanza B. Gómez ÁlvarezMonica BaroneConstanze PietschMonica BullejosConsuelo Rubio-GuerriMónica CarreraConsuelo SerresMónica CostaCoombs CassiusMónica de Los ReyesCoralia Manzanilla-PechMonica G. CandelaCoralie Danchin-BurgeMonica GiammarioliCorbin D. HillingMonica Guarino AmatoCord DrögemüllerMonica Isabella CutrignelliCorinna GrasemannMónica Lopes-MarquesCornelia MeckbachMonica M. BaqueroCorrado BattistiMonica ProboCorrado PacelliMonica RagusaCorrine LutzMónica Ramírez-MellaCosmas NathanailidesMónica Teresa González-RamírezCosme SalasMonika FajferCosmin Petru PeşteanMonika FliszkiewiczCostanza JuckerMonika GebskaCostanza RovidaMonika Hułas-StasiakCourtney HughesMonika JachCraig MichieMonika MichalczukCraig Robert ReinemeyerMonika Pogány SimonováCraig WatkinsMonika Proszkowiec-WeglarzCrisantema HernándezMonika PytkaCristián BonacicMonika Stefaniuk-SzmukierCristian LarrondoMonika Szymańska-CzerwińskaCristian Manuel Suárez-SantanaMonika WereńskaCristian MoscosoMonique BestmanCristian PirasMonique RijnkelsCristian TorresMonwadee WonglapsuwanCristiane TittoMorakot NuntapaitoonCristiano CocumelliMoreno LauraCristiano CôrtesMorgan BrisseCristina Arce JimenezMorten Dam RasmussenCristina Bonastre RafalesMorten RasmussenCristina BritoMorten TofastrudCristina CravanaMostafa Abdel-GlilCristina Esmeralda Di FrancescoMostafa S. ElshahedCristina GobelloMotoshige YasuikeCristina González-MontelongoMouad ChentoufCristina Miranda GuedesMoustafa M. ZeitounCristina PopMozaniel Santana de OliveiraCristina PornaroMrigendra RajputCristina Tomás-AlmenarMrinmoy GhoshCristina VercelliMudasir Bashir GugjooCristobal Espinosa RuizMuhammad Ali RazaCristofol PerisMuhammad Asghar HassanCrnogaj MartinaMuhammad Awais RasoolCsaba MoskatMuhammad Azher BhattiCsilla TothovaMuhammad Aziz Ur RahmanCulum BrownMuhammad BilalCynthia M. OttoMuhammad Hayat JaspalCyril RoyMuhammad IjazCyril StephenMuhammad Moaeen-Ud-DinD. Claire WathesMuhammad MohsinDac MaiMuhammad NadeemDachrit NilubolMuhammad NawazDagmar S. TrachselMuhammad Qamer ShahidDagmara NiedzielaMuhammad YousafDagmara Stępień-PyśniakMuhammed Shafeekh MuyyarikkandyDagmara WiniarczykMuhammed WalugembeDagny Krauze-GryzMuhammet KarakavukDaiji EndohMuireann ConneelyDailu GuanMujahid Ali ShahDaisuke KohariMukti BaruaDaisuke ShimizuMullakkalparambil V. SilpaDaji LuoMurat ArslanDalibor BedekovićMurat YigitDalibor TiteraMurray ItzkowitzDami MoonMurray JelinskiDamián Reyes-JáquezMusa SarıcaDamien DupontMustafa ŞahinDamien GervasoniMyanna DellingerDamien HigginsMyron ChristodoulidesDan Aaron YangMyunggi BaikDan HaoNabeel AlnahhasDan HellerNaceur M’HamdiDan Jeric Arcega RustiaNadia Andrea AndreaniDan KeremNadia MuscoDan Traian IonescuNadine BaumgartDana CampbellNadine PaßlackDana Carina SchubertNadir MamilovDanai JattawaNadja AffenzellerDanail PavlovNaduthalamuriparambu S. SudheerDanica PollardNagarajan BaskaranDaniel A. HaasNagy IstvánDaniel A. MaekawaNagy SzabolcsDaniel Acosta-AvalosNahum ShpigelDaniel AllenNaiomy D. Rios-ArceDaniel BroschartNakita CâmaraDaniel BruggerNan BaiDaniel BucurNan WuDaniel CampeloNancy de BriyneDaniel DiazNancy OguiuraDaniel FryntaNaoki MiuraDaniel García IglesiasNaoki SuzukiDaniel García SoutoNaoko TakezakiDaniel GiesekeNaomi KashiwazakiDaniel Hernandez-PatlanNaomi S. ProsserDaniel HofstetterNaomi S. TausDaniel KlichNapamanee KornthongDaniel MakowieckiNar K. GurungDaniel MarguliesNarayan DuttaDaniel MenezesNarges MashkourDaniel MierlitaNassim MoulaDaniel MillsNatalia A. ZinovievaDaniel Mota-RojasNatalia B. AnanjevaDaniel PolasikNatalia Mazur-PanasiukDaniel R. CardosoNatalia R. Dos SantosDaniel RampNatalia RyczekDaniel RiveraNatalia SiwińskaDaniel ŠpoljarićNatalia SkiepkoDaniel W. G. FörsterNatalia SowinskaDaniel WarnerNatalia V. LebedevaDániel WinklerNatalie EinDaniel Yasur-LandauNatalie WallisDaniel YorkNatascha VukasinovicDaniel ZaborskiNatascia CocchiaDaniela AlberghinaNatasha SpeightDaniela GiannettoNathalie M. H. PriymenkoDaniela IsolaNathalie PriymenkoDaniela LelesNathalie SleeckxDaniela LovarelliNathan B. SutterDaniela MoralliNausikaa DevriendtDaniela Rojas-CañizalesNavid Ghavi Hossein-ZadehDaniela Silvia PaceNayden ChakarovDaniela TakacovaNayeli Alva-MurilloDaniela WernerNazeemashahul ShamnaDaniele DendiNazrul EffendyDaniele SommaggioNcho Chris MajorDanielle Assis de FariaNedenia Bonvino StafuzzaDanijel KarolyiNegar KhayatzadehDanijela Horvatek TomicNeil WardDanijela KirovskiNektarios D. GiadinisDanila d’AngeloNele CaekebekeDanila MariniNelly OdintsovaDanilo Florentino PereiraNelson AlmeidaDannylo SousaNelson Rodrigo Da Silva MartinsDanon Clemes CardosoNemec Svete AlenkaDante JuniorNenavath Gopal NaikDanuta WrońskaNerissa RussellDany Cinq-MarsNesrein HashemDanyel Bueno DaltoNéstor SepulvedaDaoying WangNeumann Guilherme BorbaDapeng LiNey LemkeDaphne T. LianouNguyen Cong OanhDara N. OrbachNiamh CollinsDarae KangNiamh LewisDarby ProctorNiccolò FattoriniDaria MurawskaNicholas DiasDario D’OvidioNicholas Faure WalkerDariusz KokoszyńskiNicholas J. HaleyDariusz KucharczykNicholas JohnsonDariusz MikulskiNicholas K. GablerDariusz PiwczyńskiNicholas RomanoDariusz SkarzynskiNichole C. AndersonDarja PavlinNick SparksDarren MinierNico ArcillaDarryl D’SouzaNicola DecaroDarryl JonesNicola OosthuizenDavid A. WilliamsNicola PozzatoDavid AlgarNicola RooneyDavid ArgüellesNicolai CraciunDavid BowlesNicolai HildebrandtDavid CasperNicolaia IaffaldanoDavid DaviesNicolas BerthetDavid Emanuel Reyes-GuerreroNicolás GalarceDavid EnsmingerNicolas GrangerDavid FangueiroNicole Burdick SanchezDavid González-BarrioNicole GottdenkerDavid GrothNicole Pfaller-SadovskyDavid HarmonNicole R. PallottaDavid HunterNicole ReisingerDavid J. CadoganNicole VerdileDavid KilroyNicole W. XuDavid L. ThomsonNicoline SoedeDavid LalmanNicolò AmalfitanoDavid MortonNicolo ColumbanoDavid Orlando Alves FerreiraNicolò MontegioveDavid RotsteinNicolò RensiDavid S. BeggsNidia Aréchiga CeballosDavid S. FavreNieves PastorDavid S. MillerNigel B. CampbellDavid Solà-OriolNigel ReeveDavid SteffenNikhil ChauhanDavid ThompsonNiki C. WhitleyDavid Valenzuela-GalvánNikica SpremDavid W. OwensNikki KellsDavid ZapletalNikola ČobanovićDavide AgnettaNikola HodkovicováDavide AsnicarNikola KezicDavide De BiaseNikola MetodievDavide Di PaolaNikola PuvačaDavide MugettiNikolaos DiakakisDavide RondinaNikolaos VlahosDavide SoglianiNikolaus HuberDawn K. HollidayNikolina Kelava UgarkovićDawn KoltesNikos G. ChorianopoulosDawn RaultNikos PetrellisDayna DregerNils Petter KjosDean HendricksonNilusha MalmuthugeDean KonjevićNilva Kazue SakomuraDebasish Kumar DeyNima RafatiDebora DellamariaNina Čebulj-KaduncDeborah CharlesworthNina DeisigDeborah I. AdewoleNing JiaoDeborah WellsNinive Espinoza-RodriguezDebottam BhattacharjeeNipuna PereraDebra HickmanNisar AhmadDede Aulia RahmanNitin ShiroleDeepani FernandoNoah DunhamDefang ZhouNoam WernerDefu TangNobuo SasakiDehua LaiNobuyuki KanemakiDeiene Rodríguez BarretoNoelia GonzálezDeirdre P. CampionNoemí Echegaray SuárezDejan ŠkorjancNogami HirofumiDenis MuhangiNoorashikin Md NoorDenise B. O’MearaNor Azman KasanDenise Calisto BongalhardoNora M. BiermannDenise SchramaNora MestorinoDennis E. JewellNorihiko ItoDennis HeinrichNotari LorellaDennis SchmittNucharin SongsasenDerek A. RosenfieldNuno SantosDerek KnottenbeltNuno V. BritoDermeval Araújo FurtadoNuria Natalia VázquezDésirée GellatlyNuria NicodemusDesiree WandersOana-Maria BolduraDeukhwan LeeOffer ZeiraDhananjay YadavØivind BerghDhanushka N. WanasingheOleg AleksandrowiczDharmendra Kumar MeenaOleg V. TrapezovDhriti SinhaOlga FeliuDhruv DesaiOlga Jovanović GlavasDiana BellOlga LaiDiana GiannuzziOlga MitjanaDiana KoesterOlga Szalus̈-JordanowDiane BoydOlga Witkowska-PiłaszewiczDiane Elizabeth BenderOlimpia KursaDiane McVey WardOlimpia R. LaiDiane van RooyOliver Chun Hung KwokDianna BourassaOlivera ĐuragićDiederik W. van LiereOlivera MarkovićDiego FontanetoOlivier GuillaumeDiego Francisco FacconeOlivier LevionnoisDiego MartinezOlivier SparaganoDiego Rodrigo MocholiOluwatobi KolawoleDiego SustaitaOluyinka A. OlukosDieter BulachOmar Al-MassadiDietmar CrailsheimOmar Hernández-MendoDiky RamdaniOmid SafariDimas Hand Vidya ParadhiptaOndrej HanušovskýDimitar DimitrovOndrej SimonikDimitrios GougoulisOndulla T. ToomerDimitrios KanelisOphélie MenantDimitris A. GougoulisOrestis NousiasDimitris DimzasOri PomerantzDimitris K. KantasOrsola Rosa SalvaDimitris KlaoudatosOrsolya BaloghDimitris MossialosOrtega-Pacheco AntonioDing WangOsama Anwer SaeedDinithi PeirisOscar GordoDinu GavojdianOscar Ruiz-BarreraDiogo Fleury Azevedo CostaOscar VegasDiogo Francisco Maurício CoelhoOsiris GaonaDiogo Ribeiro CâmaraOskar JuhlinDionisios YoulatosOskar NilssonDirceu RamosOsman Sabri KesbiçDivino RibeiroOsmindo R. Pires JúniorDivya BeriOtilia MantaDiwakar VyasOtomar LinhartDmitrii E. AleshinOttmar DistlDmitry A. UtyanovOumou SanonDmitry VlasovOv Daniel SlaydenDoina DanesOwais A. KhanDomenico CaivanoÖzlem ÖzmenDomenico FulgioneP. Mendoza-de-GivesDomenico RobbePablo Catalá-GregoriDominga SogliaPablo DiazDominik ŁagowskiPablo E. OteroDominika GuldaPablo Jesús Marín-GarcíaDominique de ClercqPablo Luna-NevarezDominique Van der SaagPablo VergaraDominique-Marie VotionPadet SiriyasatienDomonkos VargaPagona G. GouletsouDon BradshawPajor FerencDonald C. BeitzPalmy JesudhasanDonald J. MeutenPaloma Jimena De AndrésDonata MarlettaPam CrabtreeDonatella De ZaniPam Dachung LukaDonghyun ShinPamela BurgerDonkó TamásPamela MurisonDonna RaditicPanagiota TyrnenopoulouDorgival Morais De Lima JúniorPanagiotis BerillisDoriana TedescoPanagiotis Dimitrios KatsoulosDorit KaloPanagiotis KaranisDorota BanaszewskaPanagiotis PapadopoulosDorota BoruszewskaPanagiotis SimitzisDorota Cygan-SzczegielniakPanagiotis TassisDorota JuchnoPanayiota KotsakioziDorota LewczukPanayota KoulouriDorota NowakPanchan SitthicharoenchaiDorota WitkowskaPanos PanagakisDorota WojtysiakPantu Kumar RoyDorota Zięba-PrzybylskaPaola BadinoDorothy McKeeganPaola FossatiDouglas CauseyPaola GalluzzoDouglas J. ReinemannPaola Maria ValsecchiDouglas WartzokPaola ModestoDoytcho DimovPaola RinelliDragan BrnićPaola RoncadaDragan StanojevićPaola ScanoDragana Samardžija NenadovPaola StraticòDragos ScarletPaolo AccorneroDrew LugarPaolo BaniDubravko ŠkorputPaolo BaragliDuc LuPaolo BecciuDuccio PanzaniPaolo BonilauriDudsadee MuenhorPaolo CappareDurai SellegounderPaolo ClavenzaniDuy Ngoc DoPaolo Dalla VillaDylan M. JohnsonPaolo MartelliE. M. Claudia TerlouwPaolo MerellaE. Tobias KrausePaolo PastorinoEbenezer Antwi GyameraPaolo PedataEd CharmleyPaolo PolidoriEd G. M. van KlinkPaolo PozziEdd KnowlesPaolo SilacciEdenise GarciaPaolo ViolaEdgar LehrParamanandham KrishnamoorthyEdiane ZaninPardis NajafiEdio MaldonadoPascal CrennÉdison José FassaniPascal DuenkEdivaldo Herculano Correa de OliveiraPat ShipmanEdmund MaserPatrice GaurivaudEdmundo Bonilla GonzálezPatricia A. BoleyEdo DagaroPatrícia Díaz-RosalesEdouard Reyes-GomezPatricia DiogoEdson TalaminiPatrícia Ferreira BarradasEduardo CostaPatrícia Ferreira Ponciano FerrazEduardo de MercadoPatricia HedenqvistEduardo FerreiraPatricia Martín-RodríguezEduardo Gutierrez-BlancoPatricia Mora-MedinaEduardo J. FernandezPatricia Peris-FrauEduardo José Lopes-TorresPatricia RegalEduardo José Rodríguez-RodríguezPatricia V. TurnerEduardo Marostegan PaulaPatricio R. De Los Ríos EscalanteEdward C. WebbPatrick AnselmeEdward ClaytonPatrick DescombesEdward John DickPatrick Mathews DelgadoEdward NarayanPatrick PageatEdwin Westreicher-KristenPatrick SchubertEdyta Kowalczuk-VasilevPatrizia FalabellaEdyta MakuchPatrizia NebbiaEdyta MolikPau Loke ShowEdyta PasickaPaul A. BeckEef CauwelierPaul Ayuba AbduEgon Henrique HorstPaul AzzinaroEhsan KarimiPaul BrockEiichi KanaiPaul FrickeEija KaukonenPaul HonessEinar Vargas-Bello-PérezPaul J. J. MandigersEirini FragkiadakiPaul JohnsonEkaterina MelnikovaPaul MillsEkaterina MikodinaPaul MozdziakElad LaxPaul R. KenyonElaine NortonPaul RoseElda DervishiPaul RossiterElena AlbertiPaul S. AmieuxElena BiasibettiPaul SwagemakersElena BozzettaPaul W. C. WongElena Chaves-PozoPaula Gabriela Da Silva PiresElena de FelicePaula MenziesElena González-FandosPaula MosińskaElena M. Díaz-MedinaPaula Pérez FragaElena NikitkinaPaula Toro MujicaElena RuggeriPaulina CholewińskaElena U. MartynovaPaulina JaworEleni G. KatsogiannouPaulina OpydEleni KasapidouPaulina SchmittEleni TzikaPaulo CarneiroEleonora CalzoniPaulo CortezEleonora CappellettiPaulo Fantinato NetoEleonora IaconoPaulo FavasElham PashaeiPaulo Henrique De MelloEli BaskirPavan KumarElia GattoPavel M. BorodinEliana De LucaPavel NaďEliana Ruth SteinbergPavel NevrklaElías Figueroa VillalobosPavla Manaskova-PostlerovaElias PapadopoulosPavla PostlerovaEligia Rodriguez PoncePavol ProkopElina Lastro NiñoPaweł A. KołodziejskiEline WydooghePaweł BielańskiElisa CataoPaweł BrymElisa CodecasaPaweł HanusElisa ManzocchiPaweł JaniszewskiElisa MazzottaPaweł KonieczkaElisa Pérez RamírezPaweł KordowitzkiElisa ScarsellaPaweł KowalczykElisa TorrettaPawel SokolowskiElisa WüstPaweł SolarczykElisabeth Anna Maria GraatPaweł ŻółkiewskiElisabeth PinartPayam BehzadiElisabetta ColucciaPayam DibajElisabetta CoradduzzaPeck Toung OoiElisabetta GiudicePedro FerreiraElisabetta RivaPedro Gómez-RequeniElisavet GeorgopoulouPedro GonçalvesEliseo BeldaPedro González-PechEliseo BustamantePedro Gonzalez-RedondoElisha GootwinePedro L. CastroEliza RybskaPedro Ostoa-SalomaElizabeth A. ClemmonsPedro PiñeroElizabeth A. StaigerPedro Rodríguez-LópezElizabeth BerlinerPedro SouzaElizabeth DavisPedro Vicente MichellotoElizabeth RowePedro VieiraElizabeth Watson FreemanPei-Luen LuElizabeth WoodwardPenelope BanchiElke AlbrechtPeng CuiEllen DierenfeldPeng LuoEllen Herlache-PretzerPengcheng LiEllen KienzlePenny K. RiggsEllen MartinsenPenny WatsonEllen PiercePer Moestrup JensenEllen RoelfsemaPere Miquel Parés CasanovaEllen WilliamsPerla TedescoElly HibyPerri EasonElmira MohandesanPerttu SeppaElodie WielgusPeta Lee HitchensEloy Gálvez LópezPetar KostešićElsa Leclerc DuartePeter BollenElsje PietersePeter CakebreadEluned PricePeter CoalsElvira FatsiniPeter DovčElwy AshourPeter E. BusherElżbieta BombikPeter EricksonElżbieta Hać-SzymańczukPeter F. FennessyElżbieta JancewiczPeter F. SuraiElżbieta Wnuk-PawlakPeter FordyceEm BouldPeter GrovesEman MohallalPeter H. SelleEmanuela FanelliPeter J. M. RaedtsEmanuele BerrilliPeter KnightEmanuele FasolaPeter KutzerEmanuele PanzaPeter MansellEmerson José VenancioPeter MassányiEmil BorosPeter MawsonEmilia BagnickaPeter MayoEmilia GrzędzickaPeter PaulsenEmília HanusováPeter Physick-SheardEmilia PrzygrodzkaPéter PongráczEmiliano LasagnaPeter R. MawsonEmiliano MoriPeter SandøeEmilien PelletierPeter ShurulinkovEmilio DuránPeter StathopulosEmilio HernándezPeter TozerEmilio Mármol-SánchezPeter VermeirenEmilio SabiaPetr HorákEmilio SperonePetr RauserEmily G. Patterson-KanePetr SlamaEmily J. HallPetra FrýdlováEmily J. ReppertPetra WolfEmily JohnPetras PrakasEmily N. GouldPetru Alexandru VlaicuEmily ShoesmithPhil ArkowEmir HodzicPhil GarnsworthyEmma BleachPhil HadleyEmma BucklandPhilip John LesterEmma C. CacciolaPhilip LavretskyEmma CoxPhilip RenoEmma Fàbrega i RomansPhilip SchmidtEmma MellorPhilip StephensEmma T. HelmPhilippe FravaloEmmanouil KalaitzakisPhillip E. StrydomEmmanouil MalandrakisPhillip LancasterEmmanuel A. PilaPhillip SponenbergEmmanuel Babafunso SonaiyaPhillip T. ShultsEmmanuel D. LadoukakisPhyllis ErdmanEmmanuel OkelloPier Attilio AccorsiEmmanuel RollinPier Luigi AcutisEmmanuel SerranoPier Nicola SergiEmmanuelle HaslinPiera Anna MartinoEmmanuelle TiteuxPiera MazzoneEmmelie StockPierangelo FreschiEmoke PallPierangelo MorettiEmre YemiskenPierfrancesco BiasettiEmyr PeñaPiero FranceschiEndika VarelaPiero G. GiulianiniEnikő FehérPierre ComizzoliEnnet MoholisaPierre DornyEnric GisbertPierre PicavetEnric MateuPiet J. van der AarEnrico AllevaPieter LangendijkEnrico BigliardiPietro AsproniEnrico FiorePietro LaricchiutaEnrico GugliandoloPietro LombardiEnrico LunghiPietro MedicaEnrico MancinPietro ParmaEnrico RadaelliPietro TirozziEnrico RuizPilar ForondaEnrique GómezPilar SepulvedaEnrique MejíasPin ChanjulaEnriqueta Martinez-RojasPíndaro Díaz-JaimesEraldo PassinoPing LiuErasmia SidiropoulouPinna StefaniaErdem Hüseyin InPiotr CzyzowskiEri IwataPiotr DobrowolskiEric Charles PetersonPiotr DomaradzkiEric CluaPiotr HerbutEric de Souza GilPiotr JaniszewskiEric HallermanPiotr KlimaszykEric J. ArmstrongPiotr ListosEric Lim Teik ChungPiotr OstaszewskiEric R. FethermanPiotr PerlińskiEric SaillantPiotr SkałeckiEric T. DomyanPiotr TryjanowskiEric van HeugtenPizza Ka Yee ChowEric W. DavisPo Tsang LeeErica NolPodlasińska JoannaErica ScottPol LlonchErik A. PelvePolina PerelmanErik Bø-GranquistPouya DiniErik JohnsonPrabhat Kumar PankajErik VrankenPradeepa SilvaErika JorgePragathi Belagola ShridharErika L. BergPrapansak SrisapoomeErin E. SchirtzingerPravin KaldhoneErin K. ContinoPrawej AnsariErin L. LegackiPreet SinghErin M. BrannickPriscila Arrigucci BernardesErin McConachiePriscila O. De Oliveira MoraesErin PerryPriscilla TngErnesto Gómez-FidalgoPriya DavidarEscolástico Aguilera-TejeroPriya J. HalvorsenEsmeralda DelgadoPrzemyslaw CwynarEssam M. AbdelfattahPrzemysław D. GilunEste Van Marle-KösterPrzemysław SobiechEstela MéndezPurivirojkul WatchariyaEster BartoloméPyke TinEsterina FazioQian ChenEstevam Guilherme Lux HoppeQian TangEsther IsornaQianyun XiEsther U. KadieneQin ZhaoEswar Arun KumarQing YangEto Silas FernandesQinghua LiuEttore OlmoQingsen ShangEttore VarricchioQingsong TanEuan BennetQiongxian YanEugene AndersonQiufeng ZengEugene JanzenQiuyue LiuEugenia Rita LaurianoQuanwei ZhangEugenio ScanzianiQudrat UllahEugeniusz Ryszard GrelaQuynh Anh LeEui-Chul HongR. S. A. CarlosEustachio TarascoRachael ChristensenEva ChmelíkováRachel A. HickmanEva CunhaRachel Ann DunnEva Garcia VazquezRachel C. MurrayEva JánováRachel JagoEva Mainau BrunsoRachel L. DennisEva Maria Perez MerinoRachel M. PalinskiEva PanagiotakopoulouRachel M. TaylorEva SamkovaRachel MottetEva SierraRachel WhitsedEva SpadaRachel YerburyEva TvrdáRachele AntonacciEva Varga-VisiRaciel Javier Estrada-LeónEva VítováRadhika MakechaEva VoslarovaRadiah C. MinorEva ZetterbergRadojica DjokovicEva-Maria SaliuRadomir SavićEvan AntzoulatosRadovan KasardaEvan DurlandRaed M. Al-AtiyatEvandro Maia FerreiraRafael Aguilar-GonzalezEvangelia N. SossidouRafael Fernández-MartínEvangelos G. KotsonasRafael GinésEvaristo L. MañanósRafael Julio Macedo BarragánEvaristo SouzaRafael LazzariEvelina SerriRafael MárquezEveline LitscherRafael R. GopeguiEvgeniy S. BalakirevRafael Ruiz GinésEvzen KorecRafał MartykaEwa JanuśRafał PingwaraEwa JastezębskaRafał SapierzyńskiEwa KuźnickaRaffaele BoniEwa L. GregoraszczukRaffaele MarroneEwa Olchowik-GrabarekRaffaella BranciariEwa PaździorRaffaella CoccoEwa Pecka-KielbRaffaella TudiscoEwa Sell-KubiakRahul KaulEwa Sosnówka-CzajkaRainer HuttererEwa ŚwięchRajan DhakalEwa TomaszewskaRajka BožanićEwa WójcikRalf BlankEwa ZaobibdaRalitsa BalkanskaEwa ZaobidnaRam BouchnickEwelina CzubackaRamón AriasEwelina IwanRamón ArmengolEyal SeroussiRamón CasalsEzequias Castillo LopezRamunas AntanaitisF. Abel Ponce De LeónRan DiFabiano CapparucciRandall Bruce WidelitzFábio Abade dos SantosRandall LockwoodFábio AlbuquerqueRandi BlackFabio AngeolettoRandy OliverFabio BrambillaRanga Appuhamy JayasooriyaFabio De RensisRansom BaldwinFábio MendonçaRantao ZuoFábio MeurerRaoul SchwingFabio MonticelliRaphael Kubeba TabaseFábio Pereira Leivas LeiteRaquel AtxaerandioFabrizio BertelloniRaquel DiezFabrizio CapoccioniRaquel Lama LópezFabrizio RuecaRaquel P. F. GuinéFabrizio SerenaRasa NainieneFadhlan Hafizhelmi Kamaru ZamanRasmus Bovbjerg JensenFadi HassanatRatheesh Kumar MeleppatFaith BjalobokRathnayaka Mudiyanselage Amila Subhashinie BandaraFan LiuRattanawat ChaiyaratFan YangRaul Alegria-MoranFan ZhangRaul Mainar-JaimeFares RedouaneRaúl Pérez CaballeroFarid MenaaRavesh SinghFarzad SalehpourRavi FotedarFarzana FerdousRavi P. SahuFatih SakInRavi ShahFatma EldemeryRayner Gonzalez PrendesFavián TreulénRazmaitė VioletaFazul NabiRebecca BellFederica BellagambaRebecca F. GeddesFederica BrunoRebecca ForderFederica Di CesareRebecca LanghornFederica GabbianelliRebecca MeadFederica MannelliRebecca Newcomb HomanFederica RaspaRebecca SumnerFederica RivaRebeka ZsoldosFederico ArmandoReed HolyoakFederico CalboliRegiane Rodrigues Dos SantosFederico CappaRégis SantosFederico CorlettoReid NakamuraFederico CorreaReiko NagataFederico InfascelliReiko RackwitzFederico MarroneReiner UlrichFei PengReinhard ErtlFeifei TaoReinhard ZelenyFeilong DengRemigiusz GałęckiFeiyue TengRemo LobettiFelicia MasucciRemy ManuelFelicitas AuerspergRenan Antunes DonadelliFelipe OrtegaRenan Braga PaianoFélix GoyacheRenata Navarro CassuFelix LankesterRenata RelicFélix Manuel MedinaRenato Massaaki HonjiFergus AllertonRencia van der SluisFernanda Campos de SousaRene Anne Corner-ThomasFernanda SeixasRené DörfeltFernando A. OsorioRené LacroixFernando Capela e SilvaRené Landero HernándezFernando EsperónRene St-ArnaudFernando FerreiraRené van den BromFernando Gonzalez-UarquinRenée De CrémouxFernando LaresRenyu ZhangFernando Lopez-GatiusReshma RamachandranFernando Queiroz De AlmeidaRevathi ShanmugasundaramFernando Real ValcárcelRex DunhamFernando Vargas JuniorRey David Vargas-SánchezFernando Vargas-SalinasRicardo Andrade ReisFernando Vicente MainarRicardo António Silva FariaFernando Yugo YamamotoRicardo Bárcena-GamaFestus AdejoroRicardo D. MorenoFilip TirpákRicardo F. StrefezziFilip TulisRicardo G. MaggiFilipe MadeiraRicardo GunskiFilippo FratiniRicardo J. GunskiFilippo GiarratanaRicardo Jorge LopesFilippo RossiRicardo LucenaFilippo SpadolaRicardo Perecin NocitiFiona K. HollinsheadRicardo S. RosaFiona LovattRicardo Siqueira BovendorpFiorella SarubbiRicardo Wagner PortelaFiorenzo Piccioli CappelliRicardo ZanellaFirdous A. KhanRicaurte Lopera-VásquezFlávia L. BeltrameRiccardo BozziFlavio A. DamascenoRiccardo MorettiFlavio Alves DamascenoRiccardo OrusaFlint HarrelsonRiccardo PrimiFlorbela SoaresRiccardo RinnovatiFlorence TardyRichar Rodriguez HidalgoFlorencia Di RoccoRichard A. BlatchfordFlorian BuchnerRichard A. BowenFlorian PontheauxRichard A. MansmannFlorian ZeugswetterRichard C. WatermanFlorien JennerRichard LavenFlorin MuselinRichard SeigelFlorin OanceaRichard UwieraForough AtaollahiRichard V. MachenFoteini KonstantakopoulouRichard W. HarperFouad Ali Abdullah AbdullahRick R. Van RijnFran C. SandmeierRickye S. HeffnerFrancesca ArfusoRie GotoFrancesca BeccatiRifat Ullah KhanFrancesca BennatoRikke BuhlFrancesca CarnovaleRiley ThompsonFrancesca CianiRimutu JiFrancesca ConteRina MeidanFrancesca CoppolaRina VerdiglioneFrancesca DaiRini WidayantiFrancesca DavoliRita CanipariFrancesca del SignoreRita LorenziniFrancesca FrecceroRita Payan-CarreiraFrancesca FusiRita TeodósioFrancesca GrippiRobab KataniFrancesca ManciantiRobby AnderssonFrancesca Maria SartiRobert A. CushmanFrancesca MartuzziRobert A. GitzenFrancesca MeliniRobert A. PattonFrancesca MercatiRobert BodkowskiFrancesca MossaRobert BrosnanFrancesca ParisiRobert ChleboFrancesca PirasRobert D. BradleyFrancesco BimboRobert DaileyFrancesco BordignonRobert E. MeyerFrancesco CironeRobert GiffordFrancesco CollivignarelliRobert HermesFrancesco Di IanniRobert J. CollierFrancesco FantuzRobert J. van SaunFrancesco La RussaRobert J. WalkerFrancesco M. AngeliciRobert K. BrowneFrancesco PriscoRobert KasprzakFrancesco SerrapicaRobert KissFrancesco SirtoriRobert KupczyńskiFrancesco ValerioRobert MeyerFrancine Mezzomo GiottoRobert MusundireFrancis Morais Franco NunesRobert OnzimaFrancisco A. ArrebolaRobert PasławskiFrancisco A. Leal YepesRobert RȩkawieckiFrancisco Augusto Burkert Del-PinoRobert T. KinobeFrancisco CurateRobert TwardoszFrancisco G. Véliz-DerasRoberta de Castro Souza PiaoFrancisco Javier Navas GonzálezRoberta F. GodoyFrancisco Javier Sanz-RondaRoberta FerrariFrancisco Javier Zamora-CamachoRoberta FuscoFrancisco José Nunes AntunesRoberta ImperatoreFrancisco Marlon Carneiro FeijóRoberta PecoraroFrancisco Maroto-MolinaRoberta PeregoFrancisco Miró RodríguezRoberta SalaroliFrancisco Ponce-GordoRoberta SaleriFrancisco RequenaRobertas JuodkaFrancisco RiosRoberto BaroneFranco LucchiniRoberto BertolaniFranco TagliapietraRoberto Besteiro DovalFrank CampionRoberto CaboFrank Paolo Jay B. AlbaricoRoberto ChiarelliFrank W. AbrahamsenRoberto CozzolinoFrans N. J. KooymanRoberto FrescoFranz Josef van der StaayRoberto González-GarduñoFranz SchwarzenbergerRoberto MurielFranziska KochRoberto RamoniFranziska KühneRoberto SacchiFranziska WagnerRoberto StellaFraser CombeRoberto SteriFraser HillRoberto TamburroFred SinowatzRoberto Zamora-BustillosFrederic BeugnetRobin van den BoomFrederick M. LeMieuxRobyn HudsonFrederick WilliamsRochelle Haidee YbañezFrederico VieiraRochelle StevensonFrederik H. MollenRodman GetchellFreitas VâniaRodney BrayFriederike GethöfferRodolfo Oliveira LealFriederike Katharina StockRodrigo A. AriasFriederike StumpffRodrigo FerreiraFrode KroglundRodrigo Fortunato de OliveiraFujiang HouRodrigo MorchónFukun GuiRodrigo Muiño OteroFulvia BoveraRodrigo NovaFulvio MaffucciRodrigo TorresFumikazu MatsuhisaRodrigo ValenzuelaFun-In WangRodrigues PedroFusheng SiRoger DavinG. Andres ContrerasRoger S. MorrisG. Barry BakerRogério RodriguesG. Douglas InglisRogério SilvaGabina Calderón-RoseteRoghaieh AshrafiGabriel Cipriano RochaRohana P. DassanayakeGabriel Cuevas RamosRohollah EbrahimiGabriel DallagoRoi DorGabriel Henrique de Oliveira CaetanoRoland PuschGabriela CertadRolf SchlaglothGabriela Facholi BomfimRomaan Hayat KhattakGabriela Gómez-VerduzcoRoman CroitorGabriela HarafRoman DabrowskiGabriela Knubben-SchweizerRoman NiedziółkaGabriela MotoieRomdhane RekayaGabriele BarellaRomina FusilloGabriele C. GhisleniRonak ReshamwalaGabriele MaierRonald BrooksGabriele MarinoRonald EllisGabriele MeroniRonald J. TrottaGabriele Rodrigues De LaraRonald Jan CorbeeGabriele SenczukRonald S. JonesGabriele VaccariRondineli Pavezzi BarberoGabrielle C. MuskRonick ShadrackGaby HernandezRos ClubbGaby HirsbrunnerRosa RodriguezGad DeganiRosalia FerreriGaetano CataneseRosália SáGaetano MariRosamaria LugaràGaetano OdiernaRosangela OdoreGail AndersonRosanna MarinoGail SchofieldRosaria MarinoGalia Ramírez-TolozaRosaria ScudieroGalimzhan DuskaevRosario LicitraGalina KoltsovaRosário P. Roberto da CostaGalli Gabriela MiottoRosario PitinoGarikoitz AzkonaRosaura Pérez-PeGarret SuenRose UptonGarry OsthoffRosemary HohnenGary BurrRoslyn BathgateGaspar Ros BerruezoRoss HillGautam PareekRossana CordonGayeon WonRoxana Triguero-OcañaGema Álvarez GarcíaRoxanne D. HawkinsGema Hernández MiliánRoy CostillaGema Romero MoraledaRoy DalmoGemma GonzálezRoy NagleGemma MaRoy Neville KirkwoodGemma PearsonRuan R. DarosGen KanekoRuangyote PilajunGenki KobayashiRubén Bermejo PozaGenxi ZhangRubén Guillermo PulidoGeoff FosgateRuben RosalesGeoffrey DavisonRuben SchreiterGeoffrey Wandesforde-SmithRudi Maria de MolGeona A. KimRudy Caparros MegidoGeorge AmiridisRuediger HauckGeorge F. W. HaenleinRui CostaGeorge FthenakisRui Miguel Carracha CharnecaGeorge K. SymeonRui Pedro FonsecaGeorge Lawrence PowellRunhua HanGeorge LubasRupert PalmeGeorge M. KazakosRussell S. FraserGeorge P. LaliotisRute A. CostaGeorge ValiakosRute Canejo-TeixeiraGeorgia MasonRuth Rodriguez-BermudezGeorgiana DeakRyan D. SullivanGeorgina Galicia-RosasRyan Katz-RoseneGeorgios A. GkafasRyan SamuelGeorgios ArsenosRyohei NakaoGeorgios DelisRyohei SuzukiGeorgios KarrisRyotaro MiuraGeorgios PapadopoulosRyozo TakadaGeorgios S. AmiridisRyszard TuzGeorgios TsousisRyuji HiramatsuGeorgios ValergakisRzewuska KatarzynaGerald Fritz SchusserS. AstizGéraldine BolenS. M. Lutful KabirGeraldo Tadeu Dos SantosS. R. MedeirosGerardo FatoneSabina AngreckaGercino Ferreira Virgínio JúniorSabine AbolingGergana ZahmanovaSabine KästnerGerhard KloeschSabine Schäfer-SomiGerhard SchulerSabine SchmoelzlGerhard SteenkampSabir HussainGerhard van der HorstSabrina GacemGermán EbenspergerSabrina KriefGermán M. López-IborraSacha EngelhardtGermano CastelliSachiko KoyamaGerry LushingtonSagar GoyalGerson OliveiraSage ChaiyapecharaGert VenterSahil KaliaGhader ManafiazarSaima AshrafGheorghe DărăbuşSajid Khan TahirGheorghe Mircea ConstantinescuSaki ShimamotoGholamreza Nikbakht BrujeniSalah AmashehGhulam BilalSalam A. IbrahimGhulam NabiSalim MorammaziGiacomo CremonesiSally Jane CutlerGiacomo PirloSally R. RobinsonGiada CordoniSalvador Ruiz LópezGiane Regina PaludoSalvatore BarberaGianfranco La BellaSalvatore ClapsGianluca FichiSalvatore MastrangeloGianluca NegliaSalviano Pinto SoaresGianni BattaconeSalvidio SebastianoGianniantonio DominaSamad RahimnejadGianpasquale ChiatanteSaman Abdanan MehdizadehGiedrė PiličiauskienėSamantha A. BrooksGila Abells SuttonSameer J. MabjeeshGilbert BerbenSameer PantGilles BrunschwigSami SimsekGilles FoucrasSamia BoussaaGillian L. ValeSamina AkbarGillian ScoleySamiru S. WickramasuriyaGina PinchbeckSamiya Al-RobaiyGinevra BoldrocchiSamson OladokunGioele CapilloSamuel DontaGiorgia della RoccaSamuel GinotGiorgia FabbriSamuel HockerGiorgio G. CastellaroSamuel MiñoGiorgio MasoeroSamuel MwangiGiorgio PrestiSamuel N. NahashonGiorgio ProvoloSamuel WyffelsGiorgio VignolaSanda AndreiGiovanna Lucrezia CostaSandeep JagtapGiovanna MartelliSandeep KondakalaGiovanni AmoriSandeep KumarGiovanni AsteSandeep Kumar ChavanGiovanni BuonaiutoSandor BenyheGiovanni ChillemiSandra AungierGiovanni CiliaSandra CellinaGiovanni De BenedettoSandra Costa-RouraGiovanni Della ValleSandra EdwardsGiovanni ForcinaSandra FoltinGiovanni GhibaudoSandra Gesteira CoelhoGiovanni GhielmettiSandra Goericke-PeschGiovanni LanteriSandra KuhnkeGiovanni Michele LacalandraSandra LobónGiovanni NieroSandra NicholsonGiovanni Pietro BurraiSandra RecueroGiovanni RomitoSangeeta RaoGiovanni ScillitaniSang-Hee LeeGiovanni TarantinoSanjay Kumar GuptaGisele Cristina FaveroSanjeev A. GalandeGisele MargathoSantiago Gomez SalvadorGiulia AndreaniSantiago UribeGiulia BallottaSantina Di BellaGiulia CiprianoSanto CristarellaGiulia DonadelSaqib HassanGiulia DowgierSara AlbarellaGiulia EspositoSara BarbieriGiulia FurfaroSara BusechianGiulia GuerriSara CiulliGiulia PedrettiSara Cristina Caceres RamosGiulia PignataroSara DallaresGiulia SienaSara Della TorreGiuliano GrignaschiSara FaggionGiuliano RavasioSara FontaniGiulio CuroneSara Louise CosbyGiulio Guido AiudiSara MangiaterraGiuseppe CampanileSara MuhonenGiuseppe CasconeSara NannaroneGiuseppe EspositoSara OrlowskiGiuseppe GigliaSara SechiGiuseppe ManiaciSara SuccuGiuseppe ManninoSarah Adjei-FremahGiuseppe MarruchellaSarah DoddGiuseppe MartinoSarah E. MooreyGiuseppe PiccioneSarah H. IsonGiuseppe PulinaSarah Jane HobbsGiuseppe SpinellaSarah KappelGiuseppina BasiniSarah L. LambtonGizem LeventSarah McCoskiGlayciane Costa GoisSarah MiltonGleidson Giordano Pinto De CarvalhoSarah MorrisonGleise M. SilvaSarah NaiyerGlenn van SteenkisteSarah R. B. KingGlenys NobleSarah R. T. SeidelGleyson Kleber do Amaral-SilvaSarah TalbotGloria ArriagadaSarasa MohammadiGloria CasasSarvpreet Singh GhumanGloria Cuenca-BescosSascha RohnGloria GioiaSaskia KehrausGokhlesh KumarSato ShusukeGonzalo A. ColladoSatoshi IwaseGonzalo FerreiraSatoshi KambayashiGonzalo GajardoSatoshi SekiguchiGonzalo Lopez-LorenzoSatoshi TanakaGonzalo Martínez-RodríguezSaúl Jiménez-RuizGonzalo Medina-VogelSaulius PetkevičiusGoran KišSaulo L. SilvaGoran KusecSaulo Petinatti PavariniGorana StamenkovićSauve FredericGordon BurghardtSaw BawmGordon DrydenSayed Haidar Abbas RazaGórnicki KrzysztofSayyed Mohammad Hadi AlaviGottfried HohmannSchalk CloeteGotthold GäbelScott A. DeeGoutam MondalScott A. RushGoutam SarkerScott DaviesGovind KannanScott DillonGraça LopesScott PirieGraça Maria Alexandre-PiresSe Pill ParkGraciela GarcíaSean McDonoughGraham A. McCullochSean Michael PerryGraham BurtonSean WattegederaGraham I. H. KerleySebastian Demyda PeyrásGraham MitchellSebastian HofmanGraham SmithSebastian KnagaGreg A. VicinoSebastian NowaczewskiGreg ElversSebastian OpalińskiGreg J. ComanSebastian Patrick ArltGreg S. BaxterSebastian ZeidlerGregoire LeroySebastiano BusatoGregório Miguel Ferreira De CamargoSebastiano LuridianaGregorio Ramírez ZarzosaSeham El-KassasGregory J. RobertsonSeika Hashimoto-HillGregory JohnsonSeinen ChowGregory S. FraleySejian VeerasamyGreta FoianiSelene MezzaliraGreta Veronica BerteselliSelene RubiolaGretchen GoodSeley GharaneiGrigoris AmoutziasSelim EsenGrover J. BrownSelma BeyziGrulke SigridSelma Maria Almeida-SantosGrzegorz BełżeckiSeng Fong LauGrzegorz PanasiewiczSeongjun ChoeGrzegorz WozniakowskiSerafeim ChaintoutisGrzegorz ZaleśnySerafeim PapadopoulosGrzegorz ZwierzchowskiSerdar BabacanGuadalupe Alvarez HernanSerena RicciGudrun A. BrockmannSerena SavocaGudrun StefánsdóttirSerenella D’IngeoGuido GrilliSergey OgurtsovGuido PietroluongoSergi MaicasGuido WoesteSergio Ángeles CamposGuilherme de Camargo FerrazSergio Gómez-RosalesGuilherme Henrique TamarindoSergio Iván Román-PonceGuilherme PugliesiSergio MakrakisGuillaume PéronSergio MiglioreGuillaume St-JeanSergio RochaGuillermo Alberto MattioliSergio Soto SimentalGuillermo GiovambattistaSergio Villanueva-SazGuillermo RamisSeri Intan MokhtarGuillermo Salgado MaldonadoSeunghyung LeeGuillermo Tellez-IsaiasSeungki LeeGumercindo Loriano FrancoSeveriano R. SilvaGünter GollmannSéverine HervéGuohua ZhangSe-Woon HongGuohuan SuSeyed Hossein HoseinifarGuoming LiSezayi ÖzübekGuoqing WangSezen OzkanGuoyu HuShaaban Saad ElnesrGürcan ÇetinShabana NazGuro VasdalShafqat QaisraniGustav ChládekShahid AliGustavo AmoresShahrul Anuar Mohd SahGustavo Desire Antunes GastalShaimaa SelimGustavo R. MakertShajahan FerosekhanGustavo SomozaShakeel SoomroGuy BeauchampShamsaldeen Ibrahim SaeedGuy LanzaShana BergmannGuyu QinShangzhe XieGwang Jun KimShannon Elizabeth Pratt-PhillipsGwenola Touzot-JourdeShannon GruganHa TruongShannon J. DundasHabib RehmanShannon Pratt-PhillipsHadi AtashiShaoting LiHadiar RahmanShari CohenHafez Mohamed HafezSharifah Salmah Syed-HussainHafiz Faseeh RehmanSharma SachinHafiz RizwanSharmila GhoshHai LinSharon ClouthierHailing ZongSharon CregierHaitao WangSharon GlaeserHaitao YangSharon Tirosh-LevyHaiyan SunShashwati MathurkarHaizhou LiuShau-Chi ChiHala HusseinShawn BabiukHalima SultanaShawn LarsonHamada A. M. ElwanShawna L. WeimerHamaseh TayariShedrach Benjamin PewanHamed Amirpour-NajafabadiSheena LeekHamideh KeshavarziSheila C. RahalHamutal MazrierSheila LavertyHan GongSheila Tavares NascimentoHana ŠigutováSheilah Ann RobertsonHanan Hassan Abd-ElhafeezSheng-An LeeHang YangShengguo ZhaoHani El-ZaiatShengru WuHan-Jun KimShengyu XuHanna JankowiakShengzhen HouHanna KuligShiang-Lin HuangHanna LindqvistShigeru NinomiyaHanna MoniuszkoShigeru WatanabeHannah HollingerShigetoshi YokoyamaHannah PennShihai ZhangHanne Damgaard PoulsenShih-Hsing LeirHannele KettunenShimaa AmerHans KlompenShimizu SonokoHansjörg LehnherrShin Ichiro OguraHans-Peter FuehrerShinichiro OgawaHan-Tsung WangShining GuoHany M. R. Abdel-LatifShiping BaiHanyan LiShiro MurataHao Yu ShihShiwen XuHao ZhangShogo HamaguchiHao-Kun LiuShoichi SakaguchiHao-Ting LinShota NishijimaHaoyu ShiShozo TomonagaHarald LukschShuen Ei ChenHari Prasad SharmaShuhei TakizawaHarish Kumar TiwariShuhng SunHarold C. McKenzieShu-Yin WangHarold SchottShuzhen JiangHarry DanielsShyi-Kuen YangHarry MeyerSiavash Salek ArdestaniHarun AlbayrakSiemowit MuszyńskiHasan ÖnderSigríður BjörnsdóttirHasitha Upendra PremathilakeSilas A. DavidsonHatim I. K. AlibhaiSildivane Valcácia SilvaHauck RüdigerSilva BoloñaHåvard BjørgenSilvana PietrosemoliHayford ManuSilvana PopescuHayley RandleSilvani VerruckHazel C. CarneySilvia BelliniHeather BurrowSilvia BlahakHeather FowlerSilvia CarnacciniHeather HillSilvia ColussiHeather K. MoberlySilvia DottiHeather Kristin BurleySilvia FacciniHeather M. A. FentonSilvia FerroHeba Ibrahim MohamedSilvia Gómez-SebastiánHéctor Barrios-GarridoSilvia Michela MazzolaHéctor G. Nolasco-SoriaSilvina Cecilia AndresHeidi KluessSilvio De LucaHeiko H. W. HenningSimara Marcia MarcatoHelder Patrício NunesSimo MadunaHelder QuintasSimon ClarkeHelen DaviesSimon CleggHelen LoutonSimon DanielsHelen M. GolderSimon M. TierneyHelen SchomburgSimon MoreHelen Vaterlaws-WhitesideSimon R. TewHelena ChaloupkováSimon WeigmannHélio Cordeiro Manso FilhoSimona CannasHem KatuwalSimona Kralova-KovarikovaHendrik BertschingerSimona SacchiniHeng-Lun KoSimona SagonaHengwei ChengSimona TarriconeHenk K. ParmentierSimona ValentiniHenriëtte van der ZwanSimona VincentiHenrik AndrenSimone A. BlackmanHenrik ChristensenSimone AngelucciHenrik Elvang JensenSimone BertiniHenrik LernerSimone CeccobelliHenry D. R. AlbaSimone de BrotHenry WamwayiSimone Domit GuériosHenryk KrukowskiSimone E. F. GuimarãesHerbert WagnerSimone KumstelHerman EgberinkSimone NiciuraHerminia Vergara PérezSiniša D. GrozdanićHernan BaldassarreSiobhan M. MullanHernan Diego FolcoSirje VärvHerris MaxwellSiroj PokharelHerve Hoste‪Siska AdityaHideaki TakahashiSivapong SungpraditHidehiro KondoSiyuan ZhanHideyuki MaedaSjoerd Evert Wendelaar BongaHieu Huu LeSlavi GeorgievHilary DobsonSławomir ZduńczykHilde VervaeckeSmaragda TsairidouHiroaki OkamotoSobhan AkhavanHiroaki OkawaSobhy M. A. SallamHirofumi FuruitaSofia Alves-PimentaHirohisa KishinoSofia ForssHirokazu SakamotoSofia I. GabrielHiroki OkumuraSofia LamasHiromi Takahashi-OmoeSoibam Khogen SinghHiroshi SatoSomya AggarwalHiroshi SuzukiSoňa GancarčíkováHiroyuki OkamotoSong HeHitoshi MiyasakaSonghua ShanHitoshi MurakamiSonia HerasHiva AlipourSonia Romero-RomeroHolly J. TravisSonia TassoneHolly Root-GutteridgeSonja HartnackHolly StewartSonja KoskiHong JeongwhuiSonja M. EhlersHong LiuSonja SaksidaHonggang ZhaoSonnweber RuthHongjin LiuSonsiray Alvarez-NarvaezHongjun ChenSophie GumbertHongning WangSophie RileyHongsik KongSoraia F. NevesHongyan SunSouheil GaouarHongyu MaSoumya K. KarHorst Dieter SteklisStacey SchutteHorst SpielmannStafford VigorsHossein AziziStanislaw KaminskiHoward S. BurkomStavriani KoutsouHsian-Yu WangStavros XirouchakisHsiaowei YuanStefan BauersachsHsin-I ChiangStefan PierzynowskiHsin-Yi WengStefan ProstHuafeng WangStefan SchwarzHuanca L. WilfredoStefan VilcekHuaqiang YangStefania LauziHuawei SuStefania PerrucciHubertus Van HensbergenStefanie LehnerHucheng WangStefanie P. GlaeserHugo A. BenítezStefanie PetowHugo Fernández-BellonStefanie RiemerHugo J. ParkerStefanie VeraaHugo MartinianoStefano CecchiniHui ZhangStefano CiccarelliHui-Chao YanStefano ComazziHui-Chu LinStefano GrolliHuijun GuoStefano Paolo MarelliHuiling ChenStefano RealeHuirong YangStefano RomussiHuitong ShiStefano SartoreHuitong ZhouStefano VaglioHuixian HongSteffen HoyHumberto González-MárquezStelios ZimerasHumberto González-RíosStella ChadioHung N. MaiStella de la TorreHushton BlockStella HuertasHushton C. BlockStella LeeHussein M. El-HusseinyStella Maris González CappaHuw LloydStéphane FabreHye Kwon KimStéphane JunotHyun Soon GweonStephanie A. TerryHyun Woo LeeStephanie BrittainHyungchul RahStéphanie NoëlHyungsik ShinStephen BurnettHyun-Woo KimStephen C. HardiesI. Li LiuStephen David WhiteIain McGawStephen HallIan BlandStephen M. ReedIan BouyoucosStephen NewtonIan ButtsStephen TakácsIan ForsterSteve CarverIan G. JohnstoneSteve GlasseyIan MaclachlanSteve HartIan RotherhamSteve SchapiroIan ScottSteven E. JasinskiIben MeyerSteven KoppIdoia GoiriSteven SmithIftikhar Hussain BadarStijn SchauvliegeIgnacio BadiolaStuart D. CarterIgnacio FernándezStuart GordonIgnacio GualStyliani MinoudiIgnacio Jauralde GarciaSuchana ChavanichIgnacio Ruiz-JaraboSudeb SahaIgnacy KitowskiSudheer R. BhimireddyIgo G. GuimarãesSue DysonIgor OvchinnikovSue M. McDonnellIgor SlivacSue McCoardIke OlivottoSue W. MargulisIkhide G. ImumorinSuhaili ShamsiIkuko TomoSuhel GaouarIlaria CerasoliSumali PandeyIlaria PollastriSuman Bhusan ChakrabortyIlias GiannenasSunghyun YoonIlias KarmirisSung-Jo KimIl-Jeoung YuSungsu YoukIllimar AltosaarSungwon HongIlona KaszakSungwon KimIlona SadokSupawadee PoompuangIlya A. VolodinSuresh NeethirajanIlya GordeevSureshkumar ShanmugamIlya VolodinSurita Du PreezImmaculada Armengol ArgemíSuriya RamiahImtiaz HussainSurya PaudelImtiaz RandhawaSusan C. ChapmanIn Ho KimSusan CorkIna Salwany Md YassinSusan E. ApptInder SehgalSusan HorsemanIndranil SamantaSusan J. RehorekInês Gomes CamposSusan LappanInês L. S. DelgadoSusan RobertsonInga CiprovičaSusana Amaral TeixeiraInga TiemannSusana Le BrechInge M. KrijgerSusana María Martín-OrúeInge-Marie PetzerSusana RemesarIngrid Astrid Lotta-ArevaloSusanna SalvadoriIngrid Daniëlle Ellen Van DixhoornSusanne HäußlerIngrid LorenzSusanne Hoischen-TaubnerIñigo Martínez SolanoSusanne Kreuzer-RedmerInma EstevezSushil PaudyalInmaculada Abril-ColónSusumu MitsuyamaInmaculada Martín-BurrielSusumu MuroyaInmaculada Parrilla RieraSusumu UjiInon ScharfSuyadi SuyadiInyeong KwonSuyoung JeooIoan HutuSuzanne RogersIoana Adriana MateiSven BudikIoannis A. TsakmakidisSven WuertzIoannis GiantsisSven WürtzIoannis GrivasSvenja SpringerIoannis L. OikonomidisSvetlana A. GalkinaIoannis MitsopoulosSvetlana MerenkovaIoannis SavvasSvetoslav TodorovIolanda MorettaSvjetlana Krstulović ŠifnerIon UdroiuSybille MolleIonela HoteaSydney ChertoffIppala Janardhan ReddySyed Azmal AliIrene CardinaliSyed Farhan AhmadIrene García MeilánSyed Imran Ali MeerzaIrene NoceraSyed MahmoodIrene PellegrinoSylvain FisetIrene PenarandaSylvain QuessyIrene SartiniSylvia García-BelenguerIrene ValasiSylwia ProchowskaIrenilza de Alencar NääsSylwia Żakowska-BiemansIrina BakloushinskayaSze ChanIrina Garcia IspiertoTadashi ImaiIris CharalambidouTadashi SanoIrwin Rose Alencar MenezesTadatoshi OhtakiIsa FusaroTadele KirosIsaac Hyeladi MalgwiTadeu Eder da SilvaIsaac StandishTadeusz A. JezierskiIsabel CasasTadeusz KamińskiIsabel Cristina BoleliTadeusz StefaniakIsabel MauricioTaesung JungIsabel R. DiasTaher AzimiIsabella CondottaTaichiro IshigeIsabella Corsato AlvarengaTaís Fukuta CruzIsabella Jasmine GiambraTakaharu ItamiIsabelle DufrasneTakahiro NiiIsaia SymeonidouTakanori KooriyamaIshmael Festus JajaTakaoki SaneyasuIsmael HacheroTakaomi AraiIsobel WhitingTakashi AguiIstván EgerszegiTakashi YadaIstván FodorTakayoshi UbukaIstván GyulaiTakefumi KikusuiIstván KomlósiTakeharu KanedaItai OpatovskyTakehiro NishidaIto TsuyoshiTakele ArgawItzel Amaro-EstradaTakeshi OsawaIulian Alexandru GrosuTakeshi ShimogiriIván A. García-GaliciaTakeshi YamamotoIván Hernández-CaravacaTakuo HojoIván José Galindo-CardielTali LuIvan Miguel PiresTamar GrossmanIvan MikhailovTamara P. RussoIvana ČabarkapaTamara PapovićIvelin A. MollovTamara TadichIveta PlacháTamás Gergely MolnárIvo Alexandre Leme Da CunhaTamás MolnárIvona Djurkin KušecTameka Samuels-JonesIwona JanczarekTamer AlbayrakIwona Otrocka-DomagalaTamer SharafeldinIwona PuzioTammo DelhaasIwona Rozempolska-RucińskaTanasak ChangbunjongIwona SembratowiczTanja E. WolfIwona Wojtasik-KalinowskaTanja HofmannIzabela JanusTanja M. HessIzabela RaganTanja ŠvaraIzabela SzczerbalTao LinIzabela WierzbowskaTapas K. MandalIzabela WilkTapio SolonenIzabella RządTara G. McDaneldIzhar Hyder QaziTarek MorsyJ. A. FreitasTariq JamilJ. A. RagaTariq MahmoodJ. Eduardo RicoTarja KoskelaJ. Efrén Ramírez-BribiescaTaro SugimotoJ. U. CarmonaTatiana ColchenJacek Józef NowakowskiTatjana PirmanJacek KamczycTatsuo MichiueJacek KaramonTaweepoke AngkawanishJacek KuchinkaTaylor BogarJacek NowickiTaylor Chastain GriffinJacek RechuliczTed FriendJacinto GomesTe-Hua HsuJack H. BrittTein-Shun TsaiJack J. KottwitzTemple GrandinJack ThomsonTeppei KandaJackie AbellTeresa Letra MateusJack-Yves DeschampsTeresa MogasJacob EngelmannTeresa OliveiraJacob R. TuellTeresa RochaJacopo GuccioneTeresa Semedo-LemsaddekJacopo MaffiaTeresa SouthardJacqueline BoydTereza Cristina Cardoso SilvaJacqueline MeierTerry D. BrandebourgJacques CabaretTerry EngelkenJacques FontaineTerry L. WhitingJacques RobertTerttu KatilaJaegyu YooTeruaki TozakiJagabattulla Syama DayalTeruo MaedaJagmeet KanwalTessa CarrauJagoda Kępińska-PacelikTetsumi TakahashiJai PrakashThemistoklis GiannoulisJaime BoschTheo de WaalJaime CatalanTheocharis TsoleridisJaime FigueroaTheodora PinouJaime Gallegos-SánchezTheodoros NtallarisJaime Moisés RomeroTheresa E. PancottoJaime RauTheresa L. FrankelJaime SanchisTheresa ScheuJake VeaseyThiago Pavoni Gomes ChagasJakub BabolThierry Bruno Nyatchouba NsangueJakub BiesekThiru VanniasinkamJakub GrzesiakThiruvenkadan Aranganoor KannanJakub RuszkowskiThobela NkukwanaJakub Z. KosickiThodoros E. KampourisJakyeom SeoThomas BlahaJalil Ghassemi NejadThomas Bøker LundJames BoganThomas CrenshawJames BreretonThomas D. ParsonsJames CampanellaThomas DeLibertoJames Christopher HaneyThomas EdeJames E. KinderThomas GöttertJames HillsThomas GottschalkJames K. DrackleyThomas HairgroveJames KellamThomas HartingerJames L. BonoThomas KupperJames L. KlotzThomas M. DonnellyJames LeighThomas M. GehringJames MakiThomas M. R. MaxwellJames MetcalfThomas MonseesJames Patrick CrillyThomas Oliver MéröJames R. CrabtreeThomas PüschelJames R. RussellThomas SpillmannJames SaccoThomas TullyJames WatermanThomas VannierJameson BrennanThomaz LuciaJan BeranTia G. B. HansenJan CukorTiago Antonio Del ValleJan MazurkiewiczTiago AtalaiaJan RiegertTiago BresolinJan S. SuchodolskiTiago DiasJana LendelováTiago Fernandes TavaresJana LososováTiago Marcelo OliveiraJana RadzijevskajaTiago MendonçaJana SvobodováTiago RepolhoJanaína Capelli-PeixotoTiffani Josey HowellJanaka WickramasingheTijana VučićJan-Bas PrinsTijn Jan Pieter SpoormakersJane BolesTilley PaulaJane JohnsonTim BullJane LadlowTim LukeJane M. WilliamsTim MairJane Margaret MorrellTim MorrisJane QuandtTimothy D. LeedsJane YuTimothy John MahonyJaneane IngramTina WismerJanet GenzTing LiuJanez SalobirTirawat RairatJanice E. KritchevskyTito Rafael Ferreira MendesJanice HuntingfordTiziana LiutiJanine A. DanksTobias GöckeJanine BrownTobias StraubJanka Nagyné BiróTobias WangJanne SundellTodd C. AtwoodJanne Winther ChristensenTodd CallawayJános DégiTodd FreebergJanos PostaToledo-Solís Francisco JavierJanosch ArnoldTom GearyJan-Peter BachTom HellebuyckJanusz StrychalskiTom V. SmuldersJaromir KolejkaTomas García AzcárateJaromír VašíčekTomás Landete-CastillejosJaroslav SlamečkaTomasz DaszkiewiczJarosław KrólTomasz FlorowskiJaroslaw MlynarczukTomasz HikawczukJaroslawa RutkowskaTomasz JanowskiJasmin WenderleinTomasz NiemiecJasmine FusiTomasz S. OsiejukJasmine HattabTomasz SakowskiJasmine L. LovelandTomasz SkawińskiJasminka IgrecTomasz StefanskiJasna Maršić-LučićTomasz StrzalaJason Devoy-KeeganTomasz SzablewskiJason L. MotternTomasz SzaraJason R. HerrickTomasz SzczygielskiJason RaineTomohiko KomatsuJason SawyerTomohiro YonezawaJavier AlmuniaTomokazu TakanoJavier AsinTomomi TakanoJavier Blanco-MurciaTong GuoJavier Caballero-VillalobosTong WuJavier CañónTongzuo ZhangJavier Del-Ángel-CarazaToni SafnerJavier Francisco-MorcilloTony KiszewskiJavier Gómez-ArrueToomas EsperkJavier MateoTorben StemmeJavier Morán MartínezToru TaguchiJavier Pérez-GonzálezToshie MatsumotoJavier Sánchez RomanoToshifumi MinamotoJavier Ventura-CorderoToshiharu YamamotoJaviera Ortiz-SeverínToshinori NakagawaJayasimha Rayalu DaddamToshiyoshi IchinoheJaycob D. WarfelTosso LeebJayedul HassanTracey S. ChenierJayeshbhai ChaudhariTrevor T. ZachariahJean François DohertyTristan Chalvon-DemersayJean Marie DelalandeTsewang NamgailJean Marie GraïcTuba Çiğdem OğuzoğluJean-Baptiste LecaTugay AyasanJeanette KlinkTugba GurlerJean-François PicimbonTuoying AoJean-Luc HornickTürker BodurJean-Marie ExbrayatTurner H. SwartzJean-Paul DehouxTyler A. CampbellJean-Pierre CuifTze Hann NgJean-Pierre VacherTzouramani IreneJeff WoodUğur ŞenJefferson MattosUlises Macias CruzJeffrey A. LinesUlisses Moreno-CelisJeffrey DowningUlrich ReichardJeffrey EverittUlrich SinschJeffrey OliverUlrike AuerJeffrey Robert SchmidUlrike SchererJeffrey RogersUma K. AryalJeffrey SchoenebeckUmut Sami YamakJelena RamljakUrsula M. McCormackJelena S. ĆirićUrszula LisieckaJelena ZagorskaUsman AtiqueJelka Crnobrnja-IsailovićUsman ElahiJella WautersUwe MayerJenifer WohlersUzair SajjadJennah GreenUzi MoallemJenni M. E. VirtanenV. R. FajtJennifer A. LeonardVahid MorshediJennifer B. NagashimaValbona AlikoJennifer B. SinskiValdson J. SilvaJennifer HughesValentina MonisteroJennifer K. KetzisValentina S. A. MellaJennifer L. MacNicolValentina Virginia EbaniJennifer RepacValentina ZappulliJennifer Rumsey TimmonsValentines ChisuJennifer SneddonValeria AdamovaJennifer ThomsonValeria AlbaneseJennifer Van OsValeria Anna SovranoJennifer VonkValeria BaldassarreJennifer WalkerValeria BlandaJennifer YoungValeria GriecoJennifer ZahmelValeria MaselliJenny HagenValeria MericoJenny StrackeValéria Régia Franco SousaJenő KontschánValeria SalinasJens André HammerlValeria SalvatoriJens EhmckeValeria TafintsevaJeremy D. WilsonValeria TortiJeroen DewulfValerie E. RymanJerôme PonthierValerie HughesJerry MalayerValerio SoraJerry VoleskyValeska StephanJerzy JuśkiewiczValiollah PalangiJesse ErensVan Lun LowJessica BibboVan WallachJessica BrandtVance G. NielsenJessica HillVanessa De SantisJéssica IspadaVanessa HerderJessica L. SpearsVanessa MoraesJessica Maria AbbateVanessa RabbogliattiJessica Marie da SilvaVangelis EconomouJessica MartinVânia BaptistaJessica PierceVanlata PatelJessica RomineVanner BoereJessica StorerVaros G. PetrosyanJessica Suagee BedoreVasfiye Kader-EsenJessica WernerVasia S. MavrogianniJesus A. BaroVasil PirgozlievJesús Alfredo BerdugoVasile CozmaJesús De La FuenteVasileios G. PapatsirosJesús DomínguezVasileios GreveniotisJesús HernándezVasileios PapatsirosJèsus Maria Perez JimenezVasileios V. ParaskeuasJesús Martínez-HernándezVasiliki MavrogianniJesus TomasVasilios LiordosJevrosima StevanovićVasilios TsiourisJi WangVassilios DotasJiahao PengVassilis SkampardonisJialei DuanVelmurugu RavindranJian ChengVera RarJianfeng LuVere NicolsonJiangchuan ShenVerity JonesJianglin FanVeronica CowlJiann-Chu ChenVerónica Díaz-HernándezJiann-Horng LeuVeronica R. FloresJianwei ZhouVeronica RobertsJianzhu LiuVéronique BlanquetJiatang LiVéronique OuelletJiaying HuVesna JacevicJibran HussainViatcheslav Vladimirovich RozhnovJie XuVicente Eliezer Vega-MurilloJie YangVicente Rodríguez-EstévezJieshi YuVicente Seco-RoviraJiewen GuanVicki FishlockJilan ChenVictor A. Palomino-TapiaJilei ZhangVictor Benno Meyer-RochowJill L. RichardsonVictor Breno PedrosaJim CasaerVíctor Hugo Parraguez GamboaJim DavieVictor PinheiroJimenez Carolina PalaciosVictor SpangenbergJindřich ČítekVictoria J. StrongJing ZhangVictoria K. BaxterJingbo LiuVictoria L. BoultJingguang WeiVictoria LuñoJingou TongVictoria Luño LázaroJinkoo KimVictoriano Corpas-LópezJinquan WangVida JuozaitienėJinsoo KimVidar SelasJinsu HongVijay IndukuriJinzhong TaoViju Vijayan PillaiJiqing WangVikrant SudanJirayu TanprasertsukViktar LemiasheuskiJitender DubeyViktor BrygadyrenkoJithine Jayakumar RajeswariViktoriya DzyubaJiufeng WangVilceu BordignonJixing ZouVilhjalmur SvanssonJiyun LiVinay ShivannaJo Ann ChewVincent LegrosJoachim Lübbo KleenVincent NijmanJoan M. BurkeVincenzo CarcangiuJoan Tarradas FontVincenzo CicirelliJoana CruzVincenzo CuteriJoana DamasVincenzo LandiJoana NeryVincenzo MastelloneJoana SimõesVincenzo ParrinoJoanna BarłowskaVincenzo TufarelliJoanna GŁodekVinícius De Avelar São-PedroJoanna GruszczyńskaVinícius PegoriniJoanna HockenhullVinicius R. MoreiraJoanna Jeżewska-FrąckowiakVinod R.M.T. BalasubramaniamJoanna KozłowskaVioleta JuškienėJoanna N. IzdebskaVirág ÁcsJoanna NowosadViraj MuthyeJoanna Pajdak-CzausVirgínia Alice Cruz Dos SantosJoanna Pławińska-CzarnakVirginia Celia ResconiJoanna Popiołek-KaliszVirginie DoceulJoanna RosenbergerVita Di StefanoJoanna SajewiczVita GancitanoJoanna WojtackaVitalii TimofeevJoanna ZarzynskaVito BiondiJoanne L. EdgarVito LaudadioJoanne W. H. OultramVito MartellaJoão AlmeidaVittoria BarileJoão Augusto FerreiraVittorio SaponaroJoão CardosoVivian AlmeidaJoao CostaVivian FeddernJoão CotaVivian GathJoao Henrique Moreira VianaViviana GenualdoJoão José GouveiaViviani GomesJoão OliveiraVladana GrabezJoão Paulo MedeirosVladimir DvoretskyJoão Pedro BarbasVladimir LaptikhovskyJoão QueirosVladimir PlachýJoão R. MesquitaVladimir StevanovićJoaquim Agostinho MoreiraVladimír VečeřekJoaquim BrufauVolker SchmidtJoaquim Orlando Lima CerqueiraWael A. KhalilJoaquín GadeaWafaa A. Abd El-GhanyJoaquín QuílezWagdy R. ElashmawyJoaquín SopenaWaldemar GrzegorzewskiJocelyn DubucWaleed Al-MarzooqiJochen KriegWalid AzabJodee HuntWalied AbdoJodi DeAraugoWalt E. CookJoe PaganWalter FickJoe S. SmithWalter Sánchez-SuárezJoel Bueso-RódenasWanda KrupaJoël GautronWanda OlechJohan CaratyWanilada RungrassameeJohan MichauxWare MattJohanna Rode-MargonoWarren M. SnellingJohannes Gulmann MadsenWatanya JarriyawattanachaikulJohannes HinckeldeynWattana PelyunthaJohannes Lorenz KholWawrzyniec CzubakJohannes SchenkelWei Chih LinJohn Bertram VincentWei LiangJohn Byron WilliamsWei ZhuJohn C. KilgoWeicheng ZhaoJohn CarragherWeichih LinJohn de VosWeifang ShiJohn E. GatesyWeimin WangJohn EddisonWeiping XuJohn HubbellWeisheng CaoJohn KeenWeiwei LiuJohn M. ThompsonWellison Jarles da Silva DinizJohn Marty KranabetterWenchao YangJohn MundayWendy GoodwinJohn O. OnukwuforWendy LiermannJohn P. BowmanWenjuan WangJohn P. ParkesWenjun ZhuJohn ParrishWenliang LiJohn PiltzWen-Sheng LiuJohn R. AggasWenting LiJohn R. PluskeWenwen WangJohn ReadWerner BergenJohn RodgerWerner BesseiJohn ScheibeWerner DörgelohJohn W. Dutton IIIWerner Giehl GlanznerJohn W. HarveyWerner GlanznerJohn W. KeeleWeronika MaślankoJohn WebsterWesley Lyeverton Correia RibeiroJohn Withers WalkerWiebke NeumannJolanta KomisarekWiebke SickelJolanta Krzysztoń-RussjanWiesław BielasJolanta KutkowskaWiesław SobotkaJon M. ArnemoWiesława MłodawskaJon SchoonmakerWiktor KuliczkowskiJonathan E. BeeverWilawan ThongdaJonathan ElliottWildeus StephanJonathan J. López-IslasWilfried A. KuesJonathan KoflerWilhelm GrzesiakJonathan LaMarreWill K. ReevesJong Gug KimWillem RoosenburgJongki ChoWilliam E. PinchakJonny SchoenjahnWilliam G. RyersonJordan T. GebhardtWilliam L. GainesJordana Sena LopesWilliam Martin-RossetJordi Miró RoigWilliam PérezJorge CassinelloWilliam S. LynnJorge Cerqueiro-PequeñoWilliam SmithJorge Chavez-VillalbaWillian Allan KingJorge Galindo-VillegasWinda AriyaniJorge Hugo CalvoWioletta Sawicka-ZugajJorge Moreno CharcoWitaya SuriyasathapornJorge PalenciaWitold RantJorge R. KawasWitold SzczurekJorge Ramon Lopez OlveraWlodzimierz MeissnerJorgelina Di PasqualeWojciech BarańskiJörn TheuerkaufWojciech BieleckiJörns FickelWojciech PisulaJosé A. BranWojciech ZygnerJose A. Diaz-LuqueWolfgang SiposJosé A. Pérez-MéndezWolfgang WetscherekJosé A. SilvaWon Young LeeJose A. TapiaWonchoel LeeJosé Alberto Montoya-AlonsoWoo H. KimJosé Alfredo Bran AgudeloWoolfolk SandraJose Angel Perez-AlvarezWoo-Sung KwonJose Antonio Mata-SotresWuttigrai BoonkumJosé Antonio PérezXavier AverósJosé Barbosa FilhoXavier BoivinJose C. SouzaXavier Fernández-AguilarJose CortXavier Serres-CréixamsJosé Felipe Warmling SprícigoXesús FeásJosé Fernando Machado MentenXi LiuJosé Francisco CoxXiang LiJosé Francisco PérezXiangfeng KongJosé Francisco Segura PlazaXiangjie SunJosé FresnoXianhong GuJosé Guadalupe Herrera HaroXianyao LiJosé Henrique StringhiniXiao ChenJosé Javier Santiago FreijanesXiao FeiJosé Joaquín CerónXiao HuJose L. EstradaXiao XuJosé Lino CostaXiaochuan ChenJose Louis Vega-PlaXiaodan HuangJosé Luis Granados SolerXiaojie YanJosé Luis Guzmán-GuerreroXiaojun YangJosé Luis Pérez-CastrillónXiaoling TongJosé Luis RiverosXiaorong ZhangJosé M. CarralXiaoshu ZhanJose Manuel Sanchez MorgadoXiaoxia DaiJose Maria ArroyoXiaoxue ZhangJosé María Gil-SánchezXiaoyong DuJosé Maria SantosXiaoyu LiJosé Mario MartínezXiaoyu ZhangJosé Morais Pereira FilhoXiaoyuan WeiJosé Néstor CaamañoXin LinJosé OliveiraXin LiuJose Pedro AndradeXin YangJosé Pedro AraújoXin ZhangJosé R. VillarXing LiuJosé Raduan Jaber MohamadXingping WangJosé Ramón VallejoXingsi XueJose ThekkiniathXingyi JiangJosé Yravedra-Sainz de los TerrerosXingyong ChenJosé-Daniel BarreiroXinhai LiJosef IllekXinheng ZhangJosef KameníkXinping ZhuJosep GasaXiuqi WangJosep HomedesXiuwen ZhangJosep M. Cambra BortXiyuan LuJosep PastorXóchitl Ortiz-JiménezJoseph Cyrus ParambethXu LiJoseph J. WakshlagXu WangJoseph KendraXu ZhouJoseph LozierXuan ZhuangJoseph M. BlondeauXueming ZhangJoshua Bret TaylorXueping YangJoshua D. KleinXueqing XuJoshua M. PlotnikXugang LuoJosineudson Augusto Ii Vasconcelos SilvaYadav S. BajagaiJosipa FerriYakup KaskaJoslyn BeardYamato TsujiJoy ArcherYan ZhouJoy VerrinderYanagimoto TakashiJuan Alberto CorberaYanan TianJuan Antonio Rendón HuertaYanan WangJuan Carlos Gardón PoggiYanfeng HuJuan Carlos Ku-VeraYang LinJuan DomínguezYangchun CaoJuan F. Espínola-NoveloYangdong ZhangJuan Gabriel ChediackYannis KotzamanisJuan GutierrezYanting ChenJuan Hernandez GarciaYanwen XiongJuan Hernandez MedranoYaoxin ZhangJuan José Pasantes LudeñaYasin ÜnalJuan M. Guillen-MuñozYasir IqbalJuan Manuel LomillosYasunori ShinozukaJuan Manuel PerezYasushi NumataJuan Manuel TeijeiroYauheni ZhalniarovichJuán MorgazYazavinder SinghJuan OrengoYenny RisjaniJuan P. Caldas-CuevaYeonghsiang ChengJuan P. SaucedoYeong-Hsiang ChengJuan Pedro VargasYichen ZhengJuan Peralta-SánchezYifeng ChenJuan V. González-MartínYihang LiJuan Vicente Delgado BermejoYijie XiongJuan. F. MadridYikweon JangJuan-Miguel Fregeneda-GrandesYing-Fei YangJudith Benz-SchwarzburgYingxiang LiJudith E. McDowellYiping LuoJudith Klein-SeetharamanYiwei MaJudith L. StellaYiyang ChenJudith MastersYlva Cecilia Björnsdotter SjunnessonJudith StellaYoichi MiyakeJue LiuYoichiro HoriiJuei-tang ChengYoko SatoJules DevauxYoko SattaJuli BroggiYolanda Van HeezikJulia AlbuquerqueYolande T. R. ProrogaJulia D. AlbrightYong LongJulia GonzálezYong Meng GohJulia ManorYong ZhangJulia RomanoYongfeng HeJulia WilsonYonghong ZhangJulian LangowskiYonghong ZhuJuliana RanchesYongjun ChenJuliano UczayYongliang ZhangJulie Ann WalkerYongsoo KimJulie Brastrup ClasenYongxin YangJulie NatiYoon Yeop HwangJulie OldYoonjung YiJulien DupontYordan MartínezJulien GaspariniYoshiaki HayashiJuliette YoungYoshihisa HashiguchiJúlio C. LopesYoshihito NiimuraJulio Cesar FranciscoYoshinori HatakeyamaJulio Cesar RuizYoshitaka NagamineJúlio Kuhn Da TrindadeYoshitaka NakayamaJulio Vicente Figueroa-MillánYosra A. HelmyJully Gogoi-TiwariYosra SoltanJumpei UchiyamaYosuke SasakiJun BaoYouji MaJun GongYoung Hun JinJun Hyung RyuYoung Min ChoiJun KuritaYoungjin ParkJun PengYoung-Sam KwonJuncal Gonzalez-SorianoYoung-Seuk ParkJunchul David YoonYoussef A. AttiaJunhu SuYoussef RouphaelJun-ichi ShiraishiYu LuanJunjie HanYu SunJunping ZhangYu WangJunyou LiYuan GaoJurema Corrêa Da MotaYuan LuJuriaan MetzYuan WangJurica ŽučkoYuanchun ZouJustin RichardYuanlong PanJustina PradaYuan-Tai HungJustine M. PhilipYuanzi HuoJustyna BatkowskaYubang ShenJustyna LiberaYubyeol JeonJustyna WojtaśYu-Chih WangJustyna ŻulewskaYu-Chung ChangJuvenil Enrique CaresYudai InabuJyh-Miin LinYueh-Chiang HuJyh-Mirn LaiYufeng LaiJyunsuke ShiraiYu-Hung LinK. Ann HorsburghYujiang HaoKacper LiberaYuki IwasakiKai LiuYukichika KawataKajetan Górny-KalusYu-Liang HuangKajetan PerzanowskiYulianri Rizki YanzaKálmán ImreYulong TangKamal Raj AcharyaYung-Hsiang TsaiKamil DobrzynYunlu ZhuKamil DrabikYuqing HeKamil Hakan DoğanYuri KawaguchiKamil Mert EryalçınYusheng LiangKamil SierżantYusuke HoriKamil ÜneyYutaka TakeuchiKamil WitaszekYuxin SunKamilla GrzywaczYuya NagasawaKangle YiYuzuru IkedaKannimuthu DhamotharanYvette S. Nout-LomasKaoru YoshidaZachary ShafferKara LascolaZackary A. GrahamKaraffová VieraZaira EstradaKarel NovákZamantungwa KhumaloKaren D. LupoZbigniew AdamiakKaren KindZbigniew KasprzykowskiKaren L. OverallZbigniew SobekKaren M. HarmonZdeněk KnotekKaren ThodbergZdzisław KiełbowiczKaren WimbushZebulon BellKarim El-SabroutZehe SongKarin Regina SegerŽeljko JakšićKarin WallerZengpeng LvKarin Zitterl-EglseerZengqiang ZhangKarina Guimarães RibeiroZengting LiuKarina KareninaZenon NogalskiKarine Genevieve PortierZeyang LiKarl SchedleZhao LvKarol PlesińskiZhe ZhangKarolina ArchackaZhendong ZhuKarolina ChodkowskaZhengfei LiKarolina D. JasińskaZhengwang ZhangKarolina Goździewska-HarłajczukZhenmin DingKarolina JasińskaZhibin JiKarolina LukasikZhigang ZhuKarolina WódzZhijian HuangKároly DubleczZhishuai HouKarsten DonatZhitao WangKarsten WescheZhixiong HeKarun ThongprajukaewZhixiu WangKarwan Yaseen KareemZhiyue WangKatarina Arvidsson-SegerkvistZhonghua WangKatarina MedgerZhu ZhuoKatarzyna AndraszekZhuangli KangKatarzyna BobrowiczZhuanjian LiKatarzyna Buńkowska-GawlikZiarno MałgorzataKatarzyna CzyżZicong LiKatarzyna DziewulskaZiqiang ChengKatarzyna Filip-HutschZiv Zemah-ShamirKatarzyna KirszZlatko JanječićKatarzyna KoziołZlatozar BoevKatarzyna Palińska-ŻarskaZoë CampbellKatarzyna PalusZoey DurmicKatarzyna PiórkowskaZoltán DeimKatarzyna Ropka-MolikZonghang ZhangKatarzyna ŚmiecińskaZongshuai ZhuKatarzyna TajchmanZoran LukovićKatarzyna Wojczulanis-JakubasZoran MarinovićKatarzyna ZąbekZoran StanimirovicKatarzyna ŻarczyńskaZoran VrbanacKate FarrellZorana KovacevicKate HoranZsolt GerencsérKate HutsonZsolt MaticsKate J. PlushZsolt SzendrőKate KeoghZsuzsa KalmárKate SuttonZsuzsanna SandorKatelyn MillsZulma Tatiana Ruiz-CortesKateřina FliegerováZurisaday Santos-JimenezKatharyn MitchellZuzanna MikołajczakKatherine Albro HouptZvonimir PrpićKatherine PflaumZyanya Lucía Zataraín-BarrónKatherine Vande Pol



